# Electrochemical (Bio)Sensors for Toxins, Foodborne Pathogens, Pesticides, and Antibiotics Detection: Recent Advances and Challenges in Food Analysis

**DOI:** 10.3390/bios15070468

**Published:** 2025-07-21

**Authors:** Marta Feroci, Gerardo Grasso, Roberto Dragone, Antonella Curulli

**Affiliations:** 1 Department of Basic and Applied Sciences for Engineering (SBAI), Sapienza University of Rome, via Castro Laurenziano, 7, 00161 Rome, Italy; marta.feroci@uniroma1.it; 2 Istituto per lo Studio dei Materiali Nanostrutturati (ISMN), Consiglio Nazionale delle Ricerche (CNR), Piazz.le Aldo Moro, 5, 00185 Rome, Italy; gerardo.grasso@cnr.it (G.G.); roberto.dragone@cnr.it (R.D.)

**Keywords:** food, safety, electrochemical biosensors, foodborne pathogens, toxins, pesticides, antibiotics

## Abstract

Food safety plays an important and fundamental role, primarily for human health and certainly for the food industry. In this context, developing efficient, highly sensitive, safe, inexpensive, and fast analytical methods for determining chemical and biological contaminants, such as electrochemical (bio)sensors, is crucial. The development of innovative and high-performance electrochemical (bio)sensors can significantly support food chain monitoring. In this review, we have surveyed and analyzed the latest examples of electrochemical (bio)sensors for the analysis of some common biological contaminants, such as toxins and pathogenic bacteria and chemical contaminants, such as pesticides, and antibiotics.

## 1. Introduction

Currently, food safety represents a critical and significant issue at the global scale, since it seriously affects human and animal health. Different steps during production can result in contaminants entering the food chain. For example, they can be present in food during storage and/or produced by reacting with chemical compounds. In addition, environmental contaminants, illegal adulterants, additives, and chemicals present in packaging materials are common sources of contamination in food [1,2,3].

Beyond food contamination, unhealthy or unsafe food consumption can also be triggered by food spoilage. Therefore, it is crucial to monitor every stage of the food supply chain, from the farm to table, to ensure food quality and safety [1,3]. The World Health Organization (WHO) is steadfast in its commitment to reducing the impact of foodborne diseases on global health. The WHO has led assessing the global burden of foodborne diseases and has supported the development or improvement of national surveillance systems in various countries. These efforts aim to promote national control and prevention strategies through targeted actions, including data collection, science-based risk management decision-making, educational programs, and global awareness-raising initiatives [4].

In order to preserve human health, standard-setting agencies such as the United States Food and Drug Administration (USFDA) or the European Food Safety Authority (EFSA) have established maximum limits for various contaminants in food [5,6,7].

Different types of contaminants can be present in food products due to their composition, complexity, and processing. Whether introduced intentionally or unintentionally, these contaminants can cause over 200 diseases, ranging from mild nausea to death, in both developed and developing countries, as per the WHO [4]. Four types of food contaminants can be identified: (i) chemical contaminants (e.g., mycotoxins, pesticides and herbicides, heavy metals, and industrial chemicals), (ii) allergens (e.g., milk, eggs, peanuts, and soy), (iii) biohazards (e.g., pathogenic bacteria and viruses, parasites, molds and fungi), and (iv) physical contamination (e.g., hair or fingernails, insects, microplastics, metal fragments and stones). Chemical contaminants are usually resistant to degradation and can accumulate in the body, leading to acute poisoning. Their primary sources include drugs, pesticide residues, food additives, processing-related contaminants such as acrylamide, and environmental pollutants like heavy metals and mycotoxins.

A food allergy is a type of adverse immunological response triggered by ingesting or being exposed to a food component or ingredient. Such an ingredient or food can be classified as an allergen [1,8]. In comparison, various microorganisms can cause biohazards, such as bacteria, viruses, fungi and parasites. The presence of pathogenic microorganisms in food products can occur through contaminated water and air, improper storage, and inadequate cooking temperatures.

Physical contaminants are of great concern, especially if introduced intentionally. These contaminants are usually insects and microplastics. They can derive, for instance, from packaging, pests, and poor facility maintenance, but they can induce serious problems upon ingestion.

Conventional analytical methods for detecting food safety are well documented, such as gas chromatography (GC), high-performance liquid chromatography (HPLC), gas chromatography–mass spectrometry (GC–MS), liquid chromatography–mass spectrometry (LC–MS), and enzyme-linked immunosorbent assay (ELISA) [8]. However, most of these methods involve complex procedures, high costs, long analysis times, and complex sample pretreatment, producing false-positive results.

To address or mitigate these limitations, biosensors, particularly electrochemical biosensors, have attracted considerable attention due to their simplicity, rapidity, cost-effectiveness, and portability. These biosensors are specific and responsive, and they are also appropriate for real-time monitoring and on-site analysis [1,2,3]. Recently, the literature has provided several accurate reviews illustrating all the analytical methods and techniques for food safety and introducing the (bio)sensing approach [1,2,3,8,9,10,11,12]

This review highlights the latest strategies in electrochemical biosensing for food safety, especially in detecting chemical contaminants such as antibiotics, pesticides, mycotoxins, and biohazards, including pathogenic bacteria. It covers the literature from 2022 to 2025. In particular, the sensing approaches, the electrodic materials and their role, the recognition elements required or preferred, and the related recognition mechanism and the sensor analytical performances were reported and discussed. Finally, it is crucial to consider the potential use of the sensors on real samples, compared to official validation methods, if possible.

## 2. Electrochemical (Bio)Sensors

A biosensor is considered in the literature “an integrated receptor-transducer device, which is capable of providing selective quantitative or semi-quantitative analytical information using a biological recognition element”, according to the IUPAC definition [13]. In addition, an electrochemical biosensor can be classified an “electrochemical sensor that has a biological recognition element” [14].

### 2.1. Biosensors Classification and (Bio)Recognition Element

Electrochemical biosensors are subdivided into two main groups based on the biorecognition system: biocatalytic sensors and affinity biosensors. The interaction between immobilized enzymes and the analyte can lead to a chemical change in biocatalytic sensors [1,15]. According to the literature [16], “the term affinity biosensor refers to a device incorporating immobilized biological receptor molecules that can reversibly detect receptor-ligand interactions with a high differential selectivity and in a non-destructive fashion”. Affinity biosensors are antibody-based biosensors (immunosensors), aptamer-based biosensors (aptasensors), nucleic-acid-based biosensors (genosensors), and cell-based biosensors, depending on the different biorecognition elements. Bacteriophage-based biosensors involve chemically and thermally stable virus nanoparticles as biorecognition elements [17].

The electrochemical immunosensor working principle involves transforming the result of the immunochemical reaction between the antibody and target into an electrochemical signal proportional to the target concentration. The performance of an electrochemical immunosensor is correlated to the immobilization procedure of the antibody, which should ensure the stability of the antibodies on the transducer surface, maintaining their specificity and biological activity.

Considering the aptasensors, aptamers are considered chemical antibodies that interact specifically with a target molecule, similarly to the antigen–antibody interaction. Aptamers can overcome some of the disadvantages of using antibodies as recognition elements, due to the batch variability and stability. Furthermore, aptasensors can be regenerated more easily than immunosensors because the antibodies can be irreversibly damaged with use. Aptamers and antibodies are widely used as biorecognition elements and as will be clear in this review, the choice will depend essentially on the target to be examined.

Genosensors typically involve immobilized DNA/RNA probes as recognition elements, facilitating specific hybridization reactions related to the corresponding DNA–DNA or DNA–RNA molecular recognition. Genosensors are characterized by their potential for miniaturization, sensitivity, low LODs, and simple sample pretreatment. However, the high cost and complexity of the instrumentation involved are disadvantages for the use of genosensors. More details on different features of aptasensors, immunosensors and genosensors are available in recently published reviews [1,8]

Finally, a synthetic receptor-based sensor is a chemosensor if the recognition element consists of chemical molecules or a synthetic composite [18]. For example, molecularly imprinted polymer-based (MIP) sensors are included, and the polymer, a synthetic recognition element, serves as a bioreceptor.

**Figure 1:**
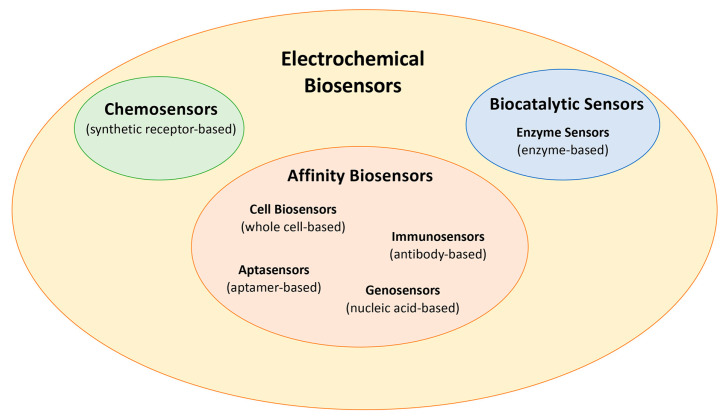
Classification of the different types of (bio)sensors.

In Figure 1, a classification of the main typologies of (bio) is shown.

In this review, we presented and discussed multiple examples of biocatalytic sensors, enzyme sensors, affinity sensors, and electrochemical chemosensors.

**Figure 2:**
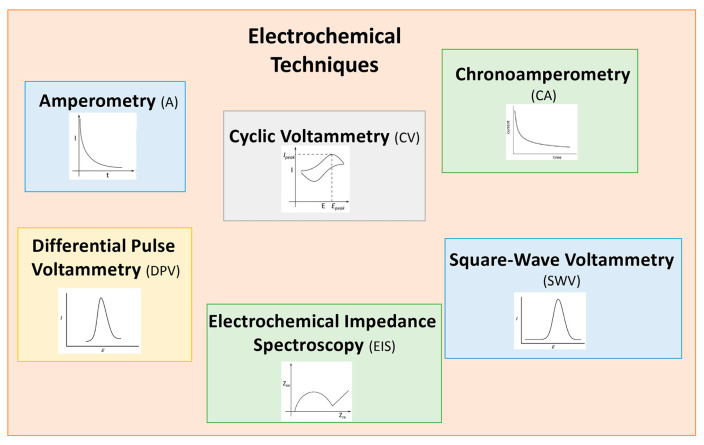
The electrochemical techniques used for biosensing applications.

Different electrochemical techniques are usually applied in the (bio)sensing area, such as amperometry (A), chronoamperometry (CA), cyclic voltammetry (CV), differential pulse voltammetry (DPV), square-wave voltammetry (SWV), and electrochemical impedance spectroscopy (EIS), as reported in Figure 2. As will be evident from Section 3, DPV, SWV, and EIS are the most widely used electrochemical techniques.

DPV and SWV are pulse voltametric techniques widely used, involving high sensitivity because the faradaic current is measured and the component of the charging current is minimized.

EIS is an important electrochemical technique that enables studying and analyzing the properties of the solution–electrode interface in correlation with the antibody–antigen immunochemical reaction, or with the target analyte–enzyme interaction, for example.

Several books and reviews in the literature are available to provide more information about the theories behind the various electrochemical techniques used for electrochemical biosensors [1,8,19,20,21,22,23].

### 2.2. Electrode Materials for Food Safety Analysis

Electrode materials, ranging from noble metals to carbon, can be used for different biosensor applications. In terms of food safety, gold and carbon are the two most common electrodic materials.

The use of gold (Au) as an electrode material in biosensors is based on its unique properties, including biocompatibility, stability, and conductivity. By modifying its surface and introducing suitable molecules, and/or nanomaterials, it is possible to improve the sensitivity and features of the Au electrode. Additionally, gold is an excellent material for microfabrication and immobilizing biomolecules.

Carbon-based electrodes include various materials, such as glassy carbon (GC) and carbon paste. The properties of conductivity, mechanical strength, renewability, and a wide potential window contribute to GC being the most commonly utilized electrodic material in biosensing. However, an accurate pretreatment procedure for GC is necessary before use [1].

Indium tin oxide (ITO) is a semiconductor of particular interest. Its transparency, ease of handling, and lower costs compared to noble metals, combined with a wider potential window than Au, make it attractive. However, ITO exhibits lower conductivity than metals and instability in acidic solutions [24,25].

Screen-printed electrodes (SPEs) are cost-effective sensing platforms, making them more attractive than conventional options. Their availability in diverse materials and geometries, both “homemade” and commercially available, enabled the development of portable analytical tools for a new generation of miniaturized biosensing devices [26,27,28].

Nanomaterials and nanostructures complement at the nanoscale level the properties of the electrodic macroscopic materials, so enhancing the electrochemical biosensor operability [29]. Nanoparticles are the most common examples of 0D nanomaterials, i.e., nanomaterials with all the dimensions in the nanoscale. Metal nanoparticles (MeNPs) were widely used. In particular, Au nanoparticles are the most widespread because of their well-known synthesis procedure, in addition to their properties, for example biocompatibility and high surface-to-volume ratio [30].

As one of the key members of nanomaterials, carbon dots (CDs) are zero-dimensional carbon nanomaterials, generally smaller than 10 nm [31,32,33]. CDs can be classified as carbon quantum dots (CQDs), graphene quantum dots (GQDs), carbon nanodots (CNDs), and carbon polymer dots (CPDs) [31,32,33,34]. Finally, CDs exhibit good biocompatibility and photoluminescence stability, and can be easily chemically modified [31,32,33,34].

Carbon nanotubes (CNTs) are the most widely studied and recognized 1D nanomaterials, i.e., a nanomaterial with two dimensions in the nanoscale. They are extensively employed in the electrochemical sensing area, as reported in the literature [29,35]. In addition, the conductivity and reactivity of CNTs are excellent, and their tubular structure and morphology facilitate their functionalization [29,35].

Carbon nanofibers (CNFs), another 1D-carbon nanomaterial, show a similar electrical conductivity to that of CNTs and conductive polymer nanofibers. Compared to CNTs, the presence of the largest edge sites on the CNFs’ outer wall can facilitate electron transfer to and from analytes. In addition, CNFs have attracted growing interest as they can be easily functionalized without affecting their structure and morphology [29].

Considering the 2D nanomaterials, they have two dimensions not within the nanoscale and present a planar morphology. In other words, they are atomically thick nanomaterials, including a single or few layers of atoms [36,37]. It is to be underlined that their mechanical and optical properties, their biocompatibility and their conductivity make these materials particularly appealing for applications in the electrochemical sensing area. Moreover, 2D nanomaterials are a large class of materials involving different elements, from transition metals to carbon, nitrogen, and/or sulfur [36]. Among them, graphene (G) is the most popular and used in different application fields, bringing attention to other 2D nanomaterials, such as MXenes, transition metal dichalcogenides (TMDs), covalent organic frameworks (COFs), and metal–organic frameworks (MOFs) [37,38,39]. Graphitic carbon nitride (g-C_3_N_4_), a novel two-dimensional (2D) semiconductor material, has attracted significant attention for applications in the fields of electrocatalysis and sensors. However, its lower conductivity can restrict electron transfer and electrocatalytic activity. To address this issue, combining g-C_3_N_4_ with other nanomaterials, such as metal or metal oxide nanoparticles, presents a potentially efficient strategy [40,41].

Finally, we consider hybrid materials: they can be regarded as a synergistic combination of various materials, including polymers, bulk materials, or nanomaterials, resulting in a composite or nanocomposite that not only enhances the properties of the original materials but can also introduce their specific and distinctive characteristics [8,42]. Exploiting and maximizing the peculiarities of the different “ingredients” in the hybrid composite enables the enhancement of the analytical performance of the resulting electrochemical biosensors. In the following section, examples of electrochemical biosensors for food safety, including diverse hybrid composites or nanocomposites, will be illustrated and discussed.

## 3. Application of Electrochemical Biosensors in Food Analysis

Electrochemical biosensors represent a significant option for determining contaminants in food, combining the sensitivity of electrochemical transducers with the specificity of the recognition element. As already indicated, several types of biosensors have been introduced, such as biocatalytic and affinity biosensors, and electrochemical chemosensors. In this review, we considered the determination of the common food contaminants such as toxins, pathogenic bacteria, pesticides and antibiotics, alongside a comparison with the conventional analytical methods, if possible.

### 3.1. Toxins

Natural toxins are biomolecules naturally synthesized by living cells or organisms (e.g., plants, bacteria, fungi, and animals) utilizing a safeguarding metabolic mechanism that protects them against serious external hazards or environmental stress factors. These toxins are potentially harmful or even lethal to humans and animals [43].

Based on the chemical structures and biological origins, natural toxins can be classified into several categories: plant-origin toxins (e.g., alkaloids, glycosides and lectins); fungal toxins (mycotoxins), such as aflatoxins, ochratoxins, trichothecenes, zearalenone and fumonisins, bacterial toxins (e.g., *Staphylococcal* enterotoxins) and marine toxins, including microcystins, tetrodotoxin, okadaic and domoic acid.

#### 3.1.1. Pyrrolizidine Alkaloids (Pas)

Beginning with plant-derived toxins, we introduced pyrrolizidine alkaloids (PAs). These compounds can be found in foods such as tea, flour, milk, honey, and meat. Consequently, long-term exposure to PAs can lead to serious human health consequences, including cytotoxicity and genotoxicity [44]. Notably, PAs with retronecine (e.g., senecionine) and heliotridine bases require extreme caution, consistent with European Food Safety Authority (EFSA) recommendations [45]. For the first time in the literature, an electrochemical chemosensor was assembled for voltametric determination of senecionine (SEN) [46]. This study investigated the influence of a DMSO/methanol solution on the sensor’s response and optimized the immobilization time of SEN on a single-use pencil graphite electrode (PGE). Differential pulse voltammetry (DPV) was employed for signal measurement. The analysis was performed without any surface functionalization, modification, or long-term electrode surface pretreatment. Under optimized conditions, the limit of detection (LOD) for SEN of 7.19 μg/mL, and a linear concentration range of 25–125 μg/mL were established. The selectivity of the sensor was investigated in the presence of intermedine, lycopsamine, and heliotropine, PAs structurally similar to SEN. Negligible changes were observed when these interferences were present. Subsequently, the reproducibility was assessed, yielding an RSD% value of 7.52. Finally, the sensor was applied to determine SEN in flour and linden tea, though the corresponding recovery data were unavailable.

#### 3.1.2. Mycotoxins

Mycotoxins, i.e., fungal toxins, are low-molecular-weight toxic secondary metabolites. The ability of numerous fungi, belonging to the genera *Aspergillus*, *Penicillium*, *Fusarium*, and *Alternaria*, to produce these toxins under similar environmental conditions often results in the co-occurrence of multiple mycotoxins in agricultural products. Mycotoxins frequently contaminate cereals, nuts, spices and dried fruits throughout their processing chain. A concerning consequence of climate change is its potential to increase mycotoxin contamination, primarily because warm and humid environments favor the proliferation of toxigenic fungi [47]. As reported in the literature [48], over 400 mycotoxins have been recognized and classified; among these, ochratoxin A (OTA), aflatoxins (AFs), zearalenone (ZEN), fumonisins (FMs), T-2 toxin (T-2), deoxynivalenol (DON) and patulin (PAT) are the most common and dangerous.

Concerning electrochemical biosensors for mycotoxins determination, detailed insights are reported in several recent reviews [49,50,51,52], including various comparisons with conventional analytical methods.

As a first example, we reported a sensor for PAT determination [53]. PAT is a polyketide lactone mycotoxin, known to contaminate fruit- and vegetable-based products, especially apples. This sensor was developed using a GCE modified with a composite of an ionic liquid-based molecularly imprinted polymer (MIP) and magnetic nanoparticles/graphene oxide (Fe_3_O_4_/GO) composite, with detection performed via SWV. Fe_3_O_4_ NPs were chosen for their non-toxicity, high surface area-to-volume ratio, catalytic activity, and chemical stability. Furthermore, the composite utilized the unique characteristics of the magnetic NPs alongside GO’s high conductivity, large surface area, and good electrochemical stability. Optimized conditions yielded a linear range of 0.001 nM to 250.0 nm. The sensor demonstrated a limit of quantification (LOQ) of 0.001 nM and a limit of detection (LOD) of 3.33 × 10^−4^ nm. The reproducibility of the sensor was investigated, resulting in a good RSD value of 0.98%. The sensor’s stability was also evaluated; after one month at 4 °C, the electrochemical signal decreased by only 1%, indicating strong long-term stability. Different organic molecules (L-cysteine, uric acid, dopamine, L-tyrosine, L-phenylalanine, aflatoxin, ochratoxin A, fumonisin B1, citric acid) and inorganic salts were tested as potential interferences. However, they did not significantly affect the peak current of PAT. The sensor was subsequently applied to spiked real samples of apple and pear juices, with recoveries ranging from 94.0% to 103.0%.

T-2 toxin is a highly cytotoxic trichothecene mycotoxin, commonly found in cereals and in cereal-based food. T-2 is a potent bioweapon agent, so specific, and rapid sensorsfor T-2 detection are required for food safety control [54].

An electrochemical microfluidic immunosensor was used to detect T-2 toxin in corn and wheat samples [55]. This sensor employed a competitive immunoassay with monoclonal anti-T-2 antibodies immobilized on a poly(methyl methacrylate) (PMMA) microfluidic channel. Its platinum wire working electrode, positioned at the end of the channel, was in situ modified through a single-step electrodeposition of a nanocomposite of reduced graphene oxide (rGO) and nanoporous gold (NPG). The competitive immunoassay relied on monoclonal antibodies T-2 immobilized on the modified electrode, where T-2 toxin in the sample competed with T-2 conjugated with horseradish peroxidase (HRP) for the antibody’s recognition sites. Optimized experimental conditions and using amperometry enabled the achievement of a linear concentration range from 0.0 to 1000 μg/kg and a LOD of 0.10 μg/kg. The precision of the immunosensor was determined using intra- and inter-assay procedures, with corresponding coefficients of variation percentage (CV%) of less than 3.98% and 5.35%, respectively. Different mycotoxins were tested to evaluate the sensor’s selectivity. The electrochemical signal was generally not affected by these interferences except for ochratoxin and deoxynivalenol. A cross-reaction was observed for these two, increasing the electrochemical response by 3% and 5%, respectively. Finally, the electrochemical sensor was applied to spiked wheat samples, and the results were compared with those obtained from ELISA, the standard conventional method. The immunosensor yielded recoveries ranging from 97.4% to 101.6%, with an inter-assay CV of less than 5.3%. In comparison, ELISA showed recoveries from 92% to 105.2%, with an inter-assay CV of less than 6.9%.

Deoxynivalenol (DON), also known as the vomiting toxin, exhibits cytotoxicity, immunotoxicity, and reproductive toxicity [56].

A portable micro-nanochannel biosensor based on 3D-printed liver microtissues was assembled for DON determination [57]. A screen-printed carbon electrode (SPCE), modified with nanoporous anodic aluminum oxide (AAO), gold nanoparticles (AuNPs), and cytochrome C oxidase (Cox), served as the working electrode. The bioink used for 3D printing contained gelatin methacrylate hydrogel and hepatocellular carcinoma cells. The resulting 3D-printed microtissue was then immobilized onto the modified electrodes. A linear detection range of 2–40 μg/mL with a LOD of 1.229 μg/mL was obtained. The selectivity, stability, reproducibility, and repeatability were not investigated. Furthermore, it was not applied to real samples, thus precluding a comparison with data from a standard reference method.

Among all mycotoxins, aflatoxins are highly toxic and carcinogenic. Several types of aflatoxins have been identified (AFB1, G1, M1, B2, etc.). Their toxicity is attributed to their chemical structure, which incorporates a difuran ring system and a coumarin moiety [58,59]. Aflatoxins are produced by the fungi belonging to the *Aspergillus* genus. Their name derived from a combination of “A” for *Aspergillus*, “fla” from flavus, (referring to its yellowish-green hue and colonies) and ‘toxins’ [58,59].

Aflatoxin B1 (AFB1) is the most toxic among aflatoxins, causing necrosis and various types of cancer. An electrochemical aptasensor for determining AFB1 was developed by electrodepositing gold nanoparticles (AuNPs) onto a GCE previously modified with zeolitic imidazolate framework-8 (ZIF-8) [60]. AuNPs exhibit a high affinity for the -SH functional group, thereby facilitating the aptamer immobilization on the Au surface via the Au-S covalent bonds. On the other hand, ZIF-8 is a very important member of MOFs, 2D nanomaterials widely employed in the electrochemical sensing field because of their easy synthetic approach, large specific surface area and chemical stability. The nanocomposite evidenced a high specific surface area, increasing the aptamer amount on the electrode surface. After the optimization of the experimental conditions and using DPV, a linear concentration range of 10.0 pg/mL–1.0 × 10^5^ pg/mL with a LOD of 1.82 pg/mL was achieved. Different aflatoxins like AFB2, AFG1 and AFG2 were used for testing the aptasensor selectivity. While AFG1 and AFG2 practically did not affect the AFB1 analysis, AFB2 slightly affected the analysis (7.3% change in signal), perhaps because AFB2 has a similar structure to AFB1. The reproducibility evidenced acceptable results in terms of RSD% (2.2%). The long-term stability was also investigated; after 15 days at 4 °C in the refrigerator, the DPV signal decreased by only 6.41%. The aptasensor was subsequently applied to spiked real samples of corn oil and peanut oil, and the results were comparable to those from ELISA. Recoveries ranged from 93.49% to 106.9% with the aptasensor, while those obtained with a commercial ELISA kit ranged from 91.84% to 99.83%.

**Figure 3:**
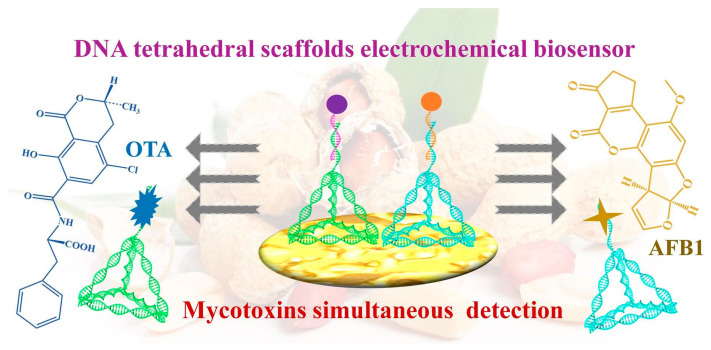
Schematic illustration of the biosensor used to determine OTA and AFB1. Reprinted with permission from [61]. Copyright 2024, Elsevier.

The next example described a bifunctional electrochemical biosensor based on DNA tetrahedral scaffolds (TDNs), for simultaneous OTA and AFB1 determination. OTA@TDNs and AFB1@TDNs were used to generate the electrochemical signal output of this combined response [61], as depicted in Figure 3.

Ochratoxin A, produced by different *Aspergillus* and *Penicillium* species, is one of the most abundant food-contaminating mycotoxins [48]. Highly porous gold (HPG) was electrodeposited on the Au electrode surface to enhance conductivity. DNA tetrahedral scaffolds (TDNs), recently introduced for biosensor assembly, are considered innovative and original DNA nanostructures possessing peculiar mechanical strength and structural stability. TDNs acted as a specific aptamer anchoring system on gold electrodes modified with HPG. OTA and AFB1 were determined through DPV. For AFB1, a linear concentration range of 0.05~360 ng/mL and a LOD of 3.5 pg/mL were found, while for OTA, a range of 0.05~420 ng/mL and a LOD of 2.4 pg/mL were observed. Zearalenone (ZEN), T-2, AFG1, and AFM1 were used as interfering molecules, and they did not affect the electrochemical response of OTA and AFB1, respectively. Regarding the bifunctional aptasensor’s reproducibility, the RSD values were 2.59% and 2.92% for OTA and AFB1, respectively. Finally, the aptasensor was applied to spiked real peanut samples, yielding recoveries between 96% and 102% for OTA and between 99% and 102% for AFB1

Zearalenone (ZEN) is classified as a xenoestrogen and resembles natural estrogens. ZEN can induce various reproductive disorders, particularly hormone imbalances, by binding to estrogen receptors. Due to its toxic nature, ZEN can result in serious diseases in humans and animals, including teratogenicity, carcinogenicity, and mutagenicity [62]. ZEN (previously known as F-2 toxin) is a non-steroidal estrogenic mycotoxin and chemically is identified as a resorcyclic acid lactone, more precisely 6-[10-hydroxy-6-oxo-trans-1-undecenyl]-B-resorcyclic acid lactone, following the IUPAC nomenclature.

An aptasensor for ZEN quantitation was developed [63], including a screen-printed electrode (SPE) and a commercial U-disk electrochemical workstation [64]. SPE was modified with a nanocomposite (AuNPs@Ce-TpBpy COF), which contained AuNPs and a covalent organic framework, specifically Ce-TpBpy COF. This COF was based on 2,2-bipyridine for building a stable framework. The aptamer was immobilized on the modified SPE through the Au–SH bond. ZEN was determined through CA, and a linear relationship with the logarithm of ZEN toxin concentration was obtained in the range of 0.001 ng/mL–10.0 ng/mL, with a LOD of 0.389 pg/mL. OTA, ochratoxin B (OTB), AFB1, aflatoxin B2 (AFB2), and fumonisins were selected as possible interfering toxins. All of them demonstrated minimal interference with the aptamer sensor response. The reproducibility was acceptable in terms of RSD (5.04%). Finally, the aptasensor was applied to spiked real samples of crops, and the recoveries ranged from 93.0% to 104.7%.

**Figure 4:**
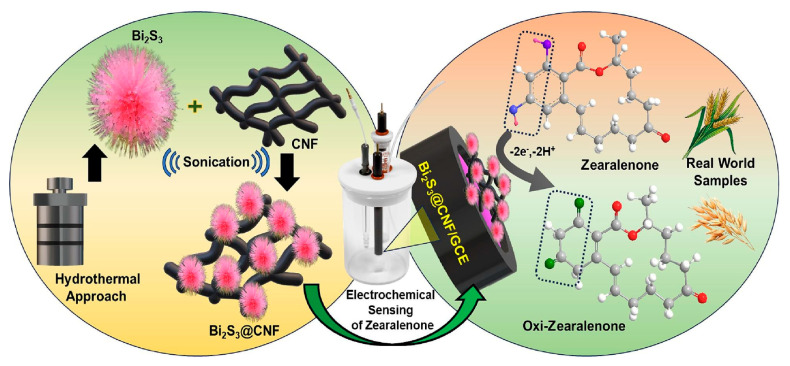
Scheme showing the process of synthesizing the Bi_2_S_3_@CNF nanocomposite and the working mechanism of the electrochemical sensor. Reprinted with permission from [65]. Copyright 2024, Elsevier.

An electrochemical sensor was assembled using Bi_2_S_3_ nanorods combined with carbon nanofibers (CNF) for ZEN detection in agricultural samples such as wheat and oats [65], as shown in Figure 4.

The resulting Bi2S_3_@CNF nanocomposite was deposited on a GCE through drop casting. ZEN was quantified via amperometry. Two linear concentration ranges were determined (0.125–375.5 and 438–1951 μM), possibly involving two different electrochemical mechanisms, with a LOD of 0.61 μM. Interfering compounds like drugs and common food additives were investigated, and the sensor’s electrochemical response was not affected by their presence. In addition, repeatability and reproducibility were analyzed, but the corresponding RSD% values were not reported. The sensor was then applied to spiked real samples of wheat and oats with recoveries ranging from 98.9% to 99.15%.

A dual-signal ratiometric electrochemical aptasensor for ZEN analysis was developed, involving MoS_2_-Thi, a hybrid chain reaction (HCR), and streptavidin-modified magnetic beads (SA-MBs) [66]. HCR represents a powerful signal amplification technique for enhancing sensor performance. MoS_2_ is a TMD semiconductor nanomaterial and was functionalized with thionine (Thi) to modify a GCE. In the presence of the analyte, SA-MBs are bound to the ZEN-apt complex, and ZEN-cDNA is released to trigger HCR. After optimizing the experimental conditions, a linear detection range (1.0 × 10^−10^–1.0 × 10^−6^ mol/L) and a LOD of 4.4 × 10^−11^ mol/L were determined, using DPV as the electrochemical technique. The selectivity was studied using OTA, AFB1, FB1 and DON, as interferences, and their presence did not significantly affect the sensor signal. Reproducibility and repeatability were investigated with satisfactory results, but the RSD% values were not provided. The aptasensor was applied to spiked real corn flour samples with recoveries ranging from 99.4% to 109.5%.

The last example of a ZEN sensor involved an electrochemical aptasensor based on the combination of functional nucleic acids, specifically aptamers and RNA-cleaving DNAzymes (FNAs) and exonuclease III-assisted target recycling (Exo III) [67]. Aptamers and RNA-cleaving DNAzymes, as functional nucleic acids, are considered to play a key role in biosensing technology for determining biomolecules. ZEN was determined through SWV, using a gold electrode modified with AuNPs as the transducer. A linear relationship between the logarithm of toxin concentration and the electrochemical current was obtained in the range of 100.0 fg/mL–50.0 ng/mL, with a LOD of 89.0 fg/mL. OTA and AFB1, together with two ZEN derivatives present in cereal products, α-zearalenone (α-ZEN) and β-zearalenone (β-ZEN), were identified as possible interfering compounds, but their presence did not significantly affect the analyte’s electrochemical response. The long-term stability was evaluated, and after 18 days at 4 °C, the signal response decreased only by 18.61%, probably because of the loss of biomolecules from the electrodic surface. The reproducibility and repeatability were tested, yielding interesting results in terms of RSD, i.e., 3.19% and 2.73%, respectively. The aptasensor was utilized to measure ZEN in spiked real samples of corn flour, peanuts, and wine. The results, with recoveries ranging from 93.52% to 110.85%, were comparable to those obtained through HPLC, which had recoveries ranging from 85.67% to 112.31%.

#### 3.1.3. Microcystins (MCs)

Microcystins (MCs) are secondary metabolites of several cyanobacteria and are present in freshwater if bacterial cells are stressed or ageing [68]. MCs are monocyclic heptapeptides including a non-proteinogenic amino acid named ADDA (3-amino-9-methoxy-2,6,8-trimethyl-10-phenyldeca-4,6-dienoic acid) and two other amino acids, generally leucine (L) and/or arginine (R). MCs are carcinogenic because they are classified as tumor promoters and trigger oxidative stress in organisms. MC-LR, the most widespread microcystin, is hepatotoxic.

An aptasensor based on a methylene blue (MB)-modified aptamer (E-AB) was assembled for detecting MC-LR [69]. The sensor’s electrochemical response is controlled by changes in the conformation and position of MB, which are caused by an interplay between the analyte and E-AB. If MC-LR is not present, the aptamer assumes a partially folded conformation. The aptamer’s interaction and binding with the analyte cause the conformation to change, facilitating the electron transfer between MB and the electrode. Consequently, the electrochemical response is improved. MC-LR was measured in the linearity range from 1.0 to 750.0 ng/L with a LOD of 0.53 ng/L, using SWV. It should be noted that this aptasensor could be regenerated and reused up to five times, after rinsing with deionized water. The MC-LR homologs, such as MC-YR and MC-RR, were chosen as possible interfering molecules. The electrochemical response to MC-LR was not significantly affected by its structural homologs. The long-term stability was evaluated, and after four weeks at 4 °C, the analyte response decreased only by 1%. Using the standard addition method, the aptasensor was applied to artificial samples of pond water and real samples of tap water, obtaining satisfactory recoveries ranging from 96.11% to 105.60%.

As the last example, we reported an immunosensor for determining MC-LR, involving a screen-printed carbon electrode modified with cysteamine, MC-LR and anti-MC-LR (anti-MC-LR/MC-LR/cysteamine/SPCE) [70]. MC-LR was measured by EIS, obtaining a linear concentration range of 0.1–100 μg/L with a LOD of 0.69 ng/L. In addition, in this case, MC-YR and MC-RR were tested as interferences, but the electrochemical response of the analyte was not affected by the presence of the MC interferents. The immunosensor was stored for 12 weeks at 4 °C the EIS response resulted in lower values than those acquired just after the sensor assembly, but the percentage decrease has not been reported. Finally, the immunosensor was used to determine MC-LR in spiked real samples of water, contaminated by cyanobacteria. MC-LR was analyzed after the bacterial cells were lysed. The results were comparable with those obtained employing ELISA, the conventional standard method.

#### 3.1.4. Marine Toxins

There are several types of toxins in the sea, usually produced by diatoms and flagellates. They accumulate in fish, crustaceans and seafood, so entering the food chain [68]. Moreover, marine toxins have high biological activity, show severe toxicity and are widely distributed throughout the world. Tetrodotoxin (TTX) is a deadly marine neurotoxin that has been identified in pufferfish, belonging to the class of Tetraodontiforms, from which the toxin takes its name [68]. TTX poisoning can cause lethargy, muscle weakness, trouble breathing, and even paralysis and death.

An electrochemical sensor based on a SPAuE modified with MIP was developed for TTX determination [71]. The MIP was synthesized via electropolymerization on the electrodic surface, and the functional monomer was o-phenylenediamine. Using DPV as an electroanalytical technique, a linear concentration range between 5.0 μg/mL and 25.0 μg/mL, with an LOD of 1.14 μg/mL was observed. The precision was evaluated using MIP sensors prepared on both the same day and different days, and the CVs were 4.9% and 13%, respectively. Glucose, together with parvalbumin (PV) and tropomyosin (TPM), allergens present in fish and seafood, were selected as possible interfering molecules, but TTX was determined without any significant interference in any case. Finally, TTX was determined in spiked mussel samples, and the corresponding recoveries were 81.0%, 110.2%, and 102.5%. They were comparable with the recoveries obtained using Hydrophilic Interaction Liquid Chromatography coupled with Tandem Mass Spectrometry, a validated method carried out by the European Reference Laboratory for Marine Biotoxins.

**Figure 5:**
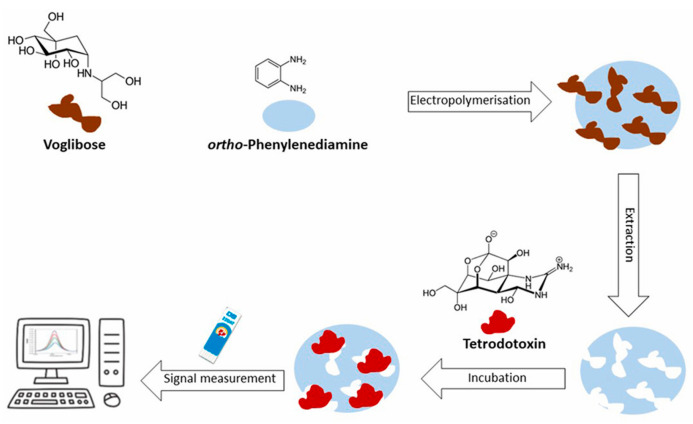
Scheme illustrating the assembly and the working mechanism of the electrochemical biosensor. Reprinted with permission from [72]. Copyright 2025, Elsevier.

Another electrochemical biosensor was realized for TTX using a specific binding peptide immobilized on polypyrrole/AuNPs-modified SPE [72], as shown in Figure 5. The bioreceptor responsible was a specific binding peptide identified through the phage display technique. The procedure of selecting the appropriate peptide with the desired affinity for the target is called biopanning [8,73].

The binding peptide was then functionalized with cysteamine to facilitate its immobilization via the Au–S bond on PPy/AuNPs/SPE. The sensing platform was prepared by successive electrodeposition of polypyrrole and AuNPs onto SPE, which improved the electrode surface conductivity and electron transfer. Using EIS, a LOD of 2.80 ppb with a detection range from 2.0 to 1000 ppb were achieved. Okadaic acid (OA), saxitoxin (STX), and domoic acid (DA) were chosen to investigate the sensor selectivity. No particular interference in the TTX determination was evidenced except in the case of STX, because of the similarity between the two toxins. The stability, reproducibility, and repeatability were not examined, and the sensor was not applied to real samples, so no comparison with data from an external standard method was provided.

Okadaic acid (OA) and its derivatives, dinophysistoxins (DTXs), are polyketide polyether lipophilic molecules. They are secondary metabolites produced by marine dinoflagellate species [68]. Okadaic acid can cause diarrhetic shellfish poisoning (DSP) in humans, leading to nausea and abdominal pain. Moreover, it affects and modifies gene expression, metabolic processes, also inducing neurotoxic, carcinogenic and genotoxic effects.

An aptasensor based on SPCE modified with chitosan (CHI) and AuNPs was prepared for the electroanalytical determination of OA [74]. The analysis was carried out by CV with a linear correlation in the range of 0.01–100 ng/mL and a LOD of 6.7 pg/mL. The selectivity was analyzed using dinophysistoxin-1 (DTX-1), dinophysistoxin-2 (DTX-2), AFB1 and MC-LR as interfering compounds, and the electrochemical signal of OA was higher than those of other toxins. Spiked real samples of mussels and scallops were analyzed utilizing the aptasensor, and satisfactory recoveries between 92.3% and 116% were found.

As conclusive comments regarding the reported examples of electrochemical biosensors for toxin detection, limits of detection (LOD) are presented in many cases in terms of pg/mL [60,61,63,74], while other examples express LOD in ng/mL [69,70] or fg/mL [67], levels that fall below those required by international standards. Unfortunately, we must point out that while data on sensor selectivity are presented accurately, data on stability, reproducibility, and repeatability are often missing. In addition, the sensors are not validated by standard or conventional methods, except for a few instances, even though it would be essential. Analytical performances of the electrochemical sensors for toxin determination, together with corresponding sensor formats, are summarized in Table 1

### 3.2. Foodborne Pathogens

Pathogenic bacteria are unicellular prokaryotic microorganisms with an average diameter of 0.5–2.0 μm and, length of 1–10 μm. They take different forms or shapes like rods (bacillus), spirals (twisted), and spheres (coccus), and they can survive and are adaptable to any environment [75]. They develop in animals, water, soil, human body, air, and food, and they induce several diseases and infections. The most common pathogenic bacteria are *Escherichia coli* (*E. coli*), *Listeria monocytogenes* (L.M.), *Staphylococcus aureus* (*S. aureus*, ST), *Salmonella* (SA), *Pseudomonas aeruginosa* (PSA), *Vibrio parahaemolyticus* (*V. parahaemolyticus*, VP) and *Campylobacter jejuni* (CJ), among others.

VP is undoubtedly one of the most significant foodborne bacteria and is connected to the bacterial gastroenteritis caused by the consumption of contaminated and infected seafood [76]. As a first example, we introduce a thread-based microfluidic electrochemical aptasensor for analyzing VP [76] The nylon threads were coated with conductive ink and functionalized with MoS_2_ nanosheets, providing a large specific surface area. MoS_2_, a 2D nanomaterial, is classified as a TMDs. Poly(lysine) (PLL) was adsorbed on the MoS_2_ nanosheets, and then the aptamer was immobilized on the MoS_2_-PLL complex via electrostatic interactions. Under the optimized experimental conditions and using DPV as an electroanalytical technique, VP was determined in the linear concentration range of 10 CFU/mL–10^6^ CFU/mL with a LOD of 5.74 CFU/mL. Next, VP was analyzed in the presence of two other pathogenic bacteria such as *E. coli* and *Enterococci*, but the DPV response of VP was not affected by the interfering bacteria. The aptasensor was applied to spiked extracts from fresh shrimps, and the results were comparable with those obtained using the conventional counting plate method.

CJ is a microaerophilic, Gram-negative foodborne bacterium commonly found in poultry that can induce severe, potentially deadly diseases in humans [77].

An impedimetric phage protein-based biosensor was developed to quantify CJ using a genetically engineered receptor-binding phage protein, FlaGrab, as a biorecognition element [77]. Biosensors based on phages and receptor-binding phage proteins (RBPPs) represent a valid alternative to genosensors and immunosensors because they are accurate and highly specific towards the target analytes [78,79]. The biosensor employed a GCE modified with MWCNTs, where the receptor was immobilized via 1-pyrenebutanoic acid, succinimidyl ester (PBSE) as molecular linker. A quasi-linear concentration range of 10^2^–10^7^ CFU/mL with a LOD of 10^3^ CFU/mL was found. The stability of the sensor was investigated, and after 15 days, the impedance had decreased to 10% of its initial value, likely due to the bioreceptor degradation over time. *Salmonella enterica* and *Listeria monocytogenes* were used as interfering bacteria, but the electrochemical response of CJ was not significantly affected by their presence.

PSA, a Gram-negative bacterium, is difficult to treat with drugs and antibiotics, resulting in serious public health concerns [80].

An innovative approach for pathogenic bacteria such as PSA can involve its quorum sensing system (QSS), which is used for cell-to-cell communication and is appropriate as a target for biosensing [80]. QSS produces molecules that are correlated with the presence of bacteria. The molecule (S)-*N*-butyryl-L-homoserine lactone (BHL) is an essential component of QSS and is assumed to be a suitable target for indirect detection of PSA. An electrochemical sensor based on an MIP-modified SPCE was prepared for the determination of BHL [80]. The polymer was synthesized via bulk free-radical polymerization using BHL as a template molecule. After the optimization of the experimental conditions and using EIS, as an electroanalytical technique, a linear range of 10−1 × 10^3^ nM and LOD of 31.78 nM were achieved. (S)-(−)-α-amino-γ-butyrolactone hydrobromide (ABL), a structural analogue to BHL, ascorbic acid, because its five-membered ring structure can be considered similar to BHL, and glucose, a common contaminant, were selected as interfering molecules. No molecules significantly affected the BHL response. The sensor was applied to spiked tap water samples, but the recovery data were not provided.

#### 3.2.1. *Staphylococcus aureus*

*Staphylococcus aureus* is a Gram-positive coccus with a spherical shape. ST adapts very well to any environment, making it commonly found in food or food residues, such as in lunch boxes, cups or glasses, as well as in infected wounds or blood [81,82].

**Figure 6:**
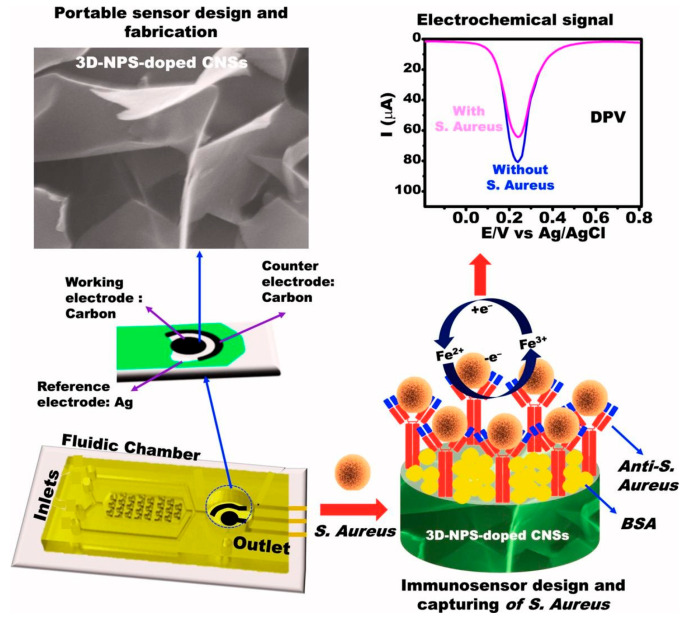
Scheme illustrating the assembly and the working mechanism of the portable immunosensor. Reprinted with permission from [83]. Copyright 2025, Elsevier.

An immunosensor based on an antibody-coated electrode with 3D multilevel micro/nano protrusions, including Au nanoclusters, was assembled for ST analysis [83], as illustrated in Figure 6. This particular electrode architecture enabled specific adsorption and effective loading of ST on the electrode.

The amount of ST was determined by EIS. The adsorption of ST induced an increase in the electrode resistance, as the bacterium is a poor conductor, which hinders charge transfer to and from the electrode. This increase in the EIS response also indicated that the antibody immobilized on the 3D multilevel electrode architecture has effectively trapped the bacterium. A linear range of 10–10^5^ CFU/mL and a LOD of 10 CFU/mL were found. *E. coli* and *Salmonella* did not affect the EIS response, indicating that the immunosensor was selective. The immunosensor was finally applied to spiked samples of milk and simulated human tissue fluid (SHTF), and the results were comparable with those obtained with the conventional counting plate method.

Next, an electrochemical biosensor based on the CRISPR/Cas9 system and rolling circle amplification (RCA)-assisted “silver chain”-linked gold interdigital electrodes (Au-IDE) was used to determine ST [84]. RCA can create long DNA chains on the Au-IDE and amplify the sensor response, supported by silver particles produced by the so-called silver staining [85]. Clustered regularly interspaced short palindromic repeats (CRISPR)/Cas9 act as a recognition component to identify/capture the target dsDNA. ST was determined by EIS, and a linear range of 10–10^5^ CFU/mL and a LOD of 7 CFU/mL were obtained. Eight different bacteria, i.e., *Escherichia coli*, *Salmonella enteritidis*, *Listeria monocytogenes*, *Clostridium dysenteriae*, *Campylobacter jejuni*, *Vibrio parahaemolyticus*, and *Bacillus cereus*, were considered as possible interferents. EIS signal changed significantly only in the presence of S. aureus in mixed samples, while it just increased if ST is absent. The reproducibility was analyzed with acceptable results in terms of RSD (2.79%). The stability was also investigated, and after 15 days at 4 °C, the electrochemical response decreased by only 7.1%.

Finally, the biosensor was applied to spiked real samples of milk, beef, fish, lettuce and bean skin with recoveries ranging from 90.63 to 113.00%. The analytical results are similar to those obtained with the conventional counting plate method.

As a further example, we would like to introduce an electrochemical genosensor that was developed for detecting ST, based on RPA-CRISPR/Cas12a, as a recognition and amplification-integrated system, utilizing a SPCE modified with a SWCNTs/AuNPs nanocomposite and hairpin DNA (hpDNA) [86]. CRISPR/Cas12a technology can recognize specific sequences of target DNA, as indicated in the previous example for CRISPR/Cas9. The combination of CRISPR/Cas12a with nucleic acid amplification techniques can accelerate the analysis process. Recombinase polymerase amplification (RPA) is a nucleic acid amplification technology that uses specific recombinases and auxiliary proteins to amplify quickly in a short time target DNA fragments. The integration of RPA and CRISPR/Cas12a technologies in an electrochemical sensor allows identifying a pathogen with precision and accuracy. ST was determined by DPV, and the corresponding linear range was 1.04 × 10–1.04 × 10^8^ CFU/mL, with a LOD of 3 CFU/mL. *Escherichia coli*, *Listeria monocytogenes*, and *Lactobacillus* were considered as possible interferents. The electrochemical response of other bacteria was merely 5% of that produced by ST. The genosensor stability was also evaluated, and after 5 days at 4 °C, the signal decreased by only 19.9%. The reproducibility data were satisfactory in terms of RSD% (3.69%).

An electrochemical aptasensor based on a nanocomposite including graphene quantum dots and Cu-MOF (GQDs/Cu-MOF) was assembled for ST detection [87]. The GQDs enhanced the sensor sensitivity and stability due to their high conductivity and functionalization. The Cu-MOF also played a role in improving the performance of the aptasensor and served as a signal label. The single-stranded DNA1 (S1) was immobilized on the surface of the modified SPCE, and the resulting S1/GQDs/Cu-MOF was considered the sensing surface. ST was detected by DPV, and a linearity range of 5.0–5.0 × 10^8^ CFU/mL with a LOD of 0.97 CFU/mL was obtained. The aptasensor stability was tested, and the sensor was stored for 30 days at 4 °C, resulting in a response decrease in less than 3.0%. Regarding reproducibility, the results are acceptable in terms of RSD (intra-assay 2.33% and inter-assay 1.98%). Different bacteria were used for testing the selectivity, such as *Y. enterocolitica, L. monocytogenes*, *Staphylococcal enterotoxin B*, *Salmonella typhimurium*, *Salmonella enteritidis*, and *Legionella pneumophila*. None of them significantly affected the ST response. Furthermore, the aptasensor was applied to detect the bacterium in spiked real samples of tap and river water, milk, *Lonicera japonica*, and urine, and the recoveries ranged from 97.30 to 106.80%. Finally, these results were comparable to those found with the ELISA conventional method.

A portable immunosensor was developed using 3D nitrogen-, phosphorus-, and sulfur-doped carbon nanosheets (3D-NPS-doped CNSs). The 3D-NPS-doped CNSs are a three-dimensional architecture with several vacant sites, a hierarchical structure, a large surface area, and rough nanosheet surfaces [88]. The heteroatoms (N, P, and S) on the carbon nanosheets surface and within the graphitic carbon network promote the surface functionalization. Consequently, several sites are present for the antibodies (anti-ST) immobilization. After the optimization of the experimental condition, using DPV, a linear relationship was obtained in the range of 1.0 × 10^2^–5.0 × 10^2^ CFU/mL, with a LOD of 24 CFU/mL. *Escherichia coli O157:H7*, *Salmonella typhimurium*, *Vibrio parahaemolyticus,* and *Shigella flexneri* were selected as possible interfering bacteria, and they did not affect the ST electrochemical response. Regarding the reproducibility, the results were satisfactory, with an RSD of 0.75%. The immunosensor retained its analytical performance even after being stored for 15 days at 4 °C. Spiked samples of water and guava juice were analyzed. The corresponding recoveries were not reported, but the results were close to those of the ELISA conventional method.

#### 3.2.2. *Listeria monocytogenes*

*Listeria monocytogenes* (L.M.), a Gram-positive bacillus, is widespread, capable of contaminating water supplies and sources, and causes an infection known as listeriosis. Pregnant women, the elderly, and individuals with immune deficiencies are particularly susceptible to this infection. To mitigate the risks of this infection, fast and accurate food analysis methods are essential [89].

As the first example, we would like to introduce an electrochemical aptasensor based on a GCE modified with silicon methylene blue (Si@MB) microspheres, gold nanoparticles (AuNPs) and a proper aptamer (Apt) [90]. Si@MB microspheres and AuNPs improved the biocompatibility and the sensitivity of the sensing interface. The aptamer was immobilized on AuNPs via Au-N bond. LM was determined by DPV and a linear concentration range from 10^2^ to 10^7^ CFU/mL, with a LOD of 2.6 CFU/mL was evidenced. *Staphylococcus aureus*, *Salmonella*, and *Escherichia coli* were selected as possible interfering bacteria, but no significant electrochemical responses were observed for any of them. For the aptasensor reproducibility, the results were satisfactory, with an RSD of 3.7%. The analysis of long-term stability yielded unsatisfactory results, likely due to the aptamer not being firmly anchored to the electrode surface and the storage conditions being inappropriate. The aptasensor was applied to spiked real samples of lettuce and fresh-cut fruits, with recoveries ranging from 84.7% to 116% for lettuce and from 80.0% to 110% for fresh-cut fruits. Finally, these results were comparable to those of the counting plate conventional method.

An electrochemical genosensor was prepared based on the integration of saltatory rolling circle amplification (SRCA) and nicking enzyme-mediated amplification (NEMA) [91]. SRCA-NEMA generated a lot of single-stranded DNA (ssDNA) through Bst DNA polymerase and Nb.BsmI, a nicking endonuclease, able to cleave only one strand of DNA on a dsDNA. DNA polymerases are enzymes that create DNA molecules by assembling nucleotides, the building blocks of DNA. In particular, Bst DNA polymerase can displace an upstream DNA strand during synthesis. For this reason and considering its thermal stability, it plays an essential role in several isothermal DNA amplification methods, including RCA. In the context of DNA, “upstream” refers to the region of the coding strand of DNA that is towards the 5′ end. This is the direction opposite to the 5′ to 3′ direction of RNA transcription and translation. The promoter, a region where RNA polymerase and transcription factors bind to initiate transcription, is typically located upstream of a gene. In the presence of LM, double-stranded DNA (dsDNA) were provided by the SRCA reaction. dsDNA could be recognized and cleaved by Nb.BsmI. The hydrosulfuryl-modified hairpin DNA (HShpDNA) was immobilized on the GCE surface, and the G-quadruplex was the signaling molecule. The linear range for LA genomic DNA was 5.4–5.4 × 10^7^ fg/μL, with the detection limit of 2.13 fg/μL. Several different bacteria were considered for the selectivity tests, but no significant electrochemical responses were observed for any of them. For the genosensor repeatability, the results were satisfactory regarding RSD (3.37%). The stability of the genosensor was analyzed by testing DPV signal changes in different electrodes. After 14 days at 4 °C, the DPV response decreased by only 9.0%. Finally, the sensor was applied to several unspecified spiked food samples. The corresponding recoveries were between 91.4% and 111.1%. In addition, the results were close to those of the conventional quantitative reverse transcription polymerase chain reaction (RT-qPCR) method.

An aptasensor was developed using a GCE modified with bimetallic silver-gold sea urchin-like hollow nanoparticles (BUHNPs) and a poly(dopamine) (PDA) film. BUHNPs were first synthesized and deposited on the electronic surface [92]. The aptamer (apt) was immobilized on the bimetallic nanoparticle structure, followed by the electrodeposition of a bacterial imprinted PDA film (PDA BIPs). L.M. was determined via DPV, following the decrease in the electrochemical signal as the bacterium concentration increased because the LA recognition prevented the electron transfer process. The concentration linear range for LA was 10^1^–10^6^ CFU/mL, with the detection limit of 1.0 CFU/mL. Concerning the long-term stability of the sensor, after 15 days at 4 °C, the DPV signal decreased by 17.2%. The reproducibility was acceptable with an RSD of 1.82%. *Escherichia coli*, *Staphylococcus aureus,* and *Bacillus subtilis* were evaluated as possible interfering bacteria, but none of them affected the LA response. The aptasensor was then used to determine LA in spiked real samples of drinking water and orange juice, with a recovery range of 90.2–105.9%.

**Figure 7:**
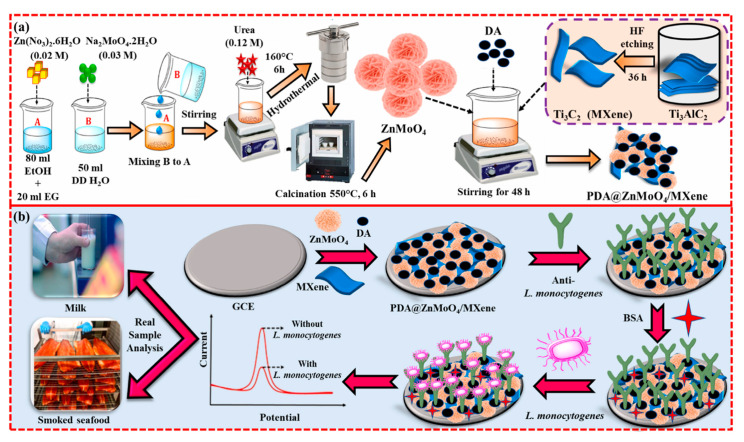
Scheme illustrating the synthesis of the PDA@ZnMoO_4_/MXene composite and the determination procedure of *Listeria monocytogenes*. Reprinted with permission from [93]. Copyright 2025, Elsevier.

As the last example for LA determination, we described a label-free electrochemical immunosensor using a mussel-inspired poly(dopamine) (PDA)-modified zinc molybdate/MXene composite (PDA@ZnMoO_4_/MXene) [93], as illustrated in Figure 7.

MXenes exhibit high electrical conductivity (rapid electron transfer), thermal stability, large surface area, hydrophilicity, and mechanical stability, making them suitable for electrochemical sensing applications. ZnMoO_4_ is an excellent biocompatible and highgly conductive electrocatalyst, with numerous active sites. PDA is a bioinspired polymer with a chemical structure similar to the marine mussel foot proteins (Mfps). It is produced through the self-polymerization of dopamine (DA), providing suitable microenvironments for the immobilization of antibodies. A linear range of 10–10^7^ CFU/mL and a limit of detection (LOD) of 12 CFU/mL were achieved using differential pulse voltammetry (DPV) as the electrochemical technique. *E. coli*, *P. aeruginosa*, *S. aureus*, and *Salmonella* were tested for selectivity, along with other molecules found in milk or meat samples such as lactose, collagen, glucose, lactic acid, and casein. The results indicated that a significant change in the DPV response was observed only in the presence of lactic acid due to the formation of antigen–antibody immune complexes. The parameters of repeatability, reproducibility, and stability were investigated. For both reproducibility and repeatability, changes in the electrochemical response were not significant, although the corresponding relative standard deviation percentages were not provided. After storing for 10 days at 4 °C, the DPV response decreased by 20.3%, likely due to the detachment of antibodies from the electrode surface and their gradual loss of activity. The applicability of the immunosensor was assessed using spiked real samples of milk and smoked seafood. The resulting recoveries ranged from 98% to 126% for milk and from 99.7% to 117% for smoked seafood.

#### 3.2.3. *Salmonella*

*Salmonella,* belonging to the family *Enterobacteriaceae*, is a Gram-negative, rod-shaped bacterium. Pathogenic species include over 2500 serotypes, such as *Salmonella Typhimurium* and *Salmonella Enteritidis* [94]. Salmonellosis can be caused by these two serotypes, and the symptoms include diarrhea, fever, and vomiting. Furthermore, *Salmonella Typhimurium*-induced typhoid fever is a life-threatening condition that can also result in death [94].

A conductive plastibody-based electrochemical sensor was prepared for the determination of *Salmonella typhimurium* [95]. The conductive plastibody was realized by modifying an ITO surface electrode through the electrodeposition of a blend including pyrrole, lactic acid, ammonium chloride, and sodium dodecyl sulfate. The plastibody was imprinted with the bacterium and can be assumed as an MIP. Amperometry was used as the electrochemical analytical technique. A linear concentration range of 100–108 CFU/mL, with a limit of detection of 3.42 CFU/mL was found. The reproducibility was evaluated using five sets of plastibody sensors, and the corresponding amperometric responses were analyzed in terms of RSD% (0.11%). *Escherichia coli*, *Staphylococcus aureus* and *Listeria monocytogenes* were considered for the selectivity tests, but their response was not significant. The plastibody sensor was applied to spiked real samples of water with recoveries ranging from 96.94% to 108.25%.

An electrochemical genosensor was developed to detect *S. typhimurium* employing a combination of polymerase chain reaction (PCR), as the amplifying system and CRISPR/Cas12a [96]. CRISPR/Cas12a technology can recognize specific sequences of target DNA, as indicated in the previous examples [84,86]. Moreover, hairpin DNA (hpDNA), acting as a signaling system, immobilized on AuE, is utilized to improve the electron transfer rate. Under the optimized conditions, a linear range from 6.7 × 10^1^ to 6.7 × 10^5^ CFU/mL with LOD of 55 CFU/mL was obtained by DPV. *E. coli* O157:H7, *V. parahaemolyticus, L. monocytogenes*, and *S. aureus* were used for the selectivity. Their DPV response was comparable to the blank sample. The genosensor was used to analyze spiked real samples of poultry, but no recovery data were provided.

A DNA electrochemical sensor was assembled based on a GCE modified with a nanocomposite comprising chitosan, CNTs, and MoS_2_, a TMD nanomaterial previously introduced [66,76], and ssDNA for detecting *S. Typhimurium* [97]. After the optimization of the experimental conditions and using DPV, a linearity range of 0.01 aM^−1^ μM and a LOD of 0.01 aM were reached, with an acceptable reproducibility of 0.62% (RSD%). Selectivity was addressed using different base mismatches, target DNA sequences, and DNA sequences of other pathogens. No significant difference in DPV responses was evidenced for the pathogenic bacteria selected compared to the blank control. The genosensor stability was investigated by storing it at 4 °C for 20 days, and the DPV signal remained unchanged. The reproducibility was evaluated, and the corresponding electrochemical responses regarding RSD% (0.62%) were analyzed. Recovery tests were performed in spiked water and milk samples, and the corresponding recoveries were from 92.95% to 99.58%.

An electrochemical biosensor was developed using bacterial cellulose (BC) modified with PPY and rGO, and functionalized with immobilized *S. typhimurium*-specific phage particles [98]. The BC substrate has a fibrous and porous structure, modified with PPY ì polymerized in the presence of RGO. The resulting biointerface was highly conductive and flexible. The immobilization of phage particles was supported by electrostatic interactions between the positively charged PPy and the negatively charged phage particles. The biointerface served as a working electrode, and *S. typhimurium* was determined by DPV, obtaining a linear concentration range of 1–10^7^ CFU/mL and a LOD of 1.0 CFU/mL. The biosensor selectivity and specificity were addressed using live and dead bacterial cells, and the DPV response to non-specific bacteria was insignificant compared to that of the *S. typhimurium.* The authors reported that the reproducibility was satisfactory, but the RSD% value was not indicated. The biosensor was considered stable because after five weeks at 4 °C, the DPV signal remained virtually unchanged. Recovery tests were conducted in spiked beef and milk samples, yielding corresponding recoveries of 100.3% and 100.4%, respectively. The obtained data were comparable to those from the conventional counting plate method.

We consider, as the next example, an electrochemical immunosensor based on a GCE modified with Fe_3_O_4_@Prussian blue (PB) core–shell nanomaterial, prepared via the PB in situ synthesis protocol on the Fe_3_O_4_ surface through acid etching [99]. The antibody was then immobilized on the nanocomposite using glutaraldehyde as a cross-linker. The dual signal amplification strategy involved Fe_3_O_4_@PB as the internal signal and [Fe(CN)_6_]^3−/4−^ ionic redox probe as the external signal. *S. Typhimurium* was determined by DPV, obtaining a linear concentration range of 7.375 × 10 to 7.375 × 10^7^ CFU/mL, with a detection limit of 9.912 CFU/mL. *Pseudomonas aeruginosa*, *Escherichia coli O157:H7*, *Proteus bacillus vulgaris*, *Shigella sonnei*, *Staphylococcus aureus* and *Listeria monocytogenes* were identified as interfering bacteria. The interfering bacteria’s responses were negligible if compared with those of *S. typhimurium*. The reproducibility was evaluated using five immunosensors, and a satisfactory RSD value was achieved (2.18%). The biosensor was considered quite stable because after 15 days at 4 °C since the DPV signal decreased of 20.18%. Finally, the immunosensor was applied to spiked milk samples, and the corresponding recoveries ranged from 99.74% to 106.40%.

As the next example, we report an electrochemical aptasensor for *S. typhimurium* detection based on poly(xanthurenic acid) (PXA) and chondroitin sulfate (CS) because of its antifouling capability [100]. CS is a biocompatible and non-toxic heteropolysaccharide belonging to the class of glycosaminoglycans and contains several carboxyl (-COOH), amide (-CONH) and hydroxyl (-OH) groups. CS has strong proton acceptor properties and a high hydration strength due to the presence of these functional groups [100]. Xanthurenic acid (XA) is a molecule of the tryptophan NAD pathway. Its polymer PXA exhibits high redox activity and low toxicity and has been used for determining biomolecules [101,102].

PXA was electrosynthesized on GCE. Subsequently, CS was covalently linked via its carboxyl groups to a PDA layer, which was electrodeposited on the PXA film, to provide antifouling properties to the surface. Finally, the aptamer of S. typhimurium was immobilized on the modified GCE. *S. typhimurium* was detected by DPV, indicated by the peak current decrease as the bacterium was trapped on the electrode surface, preventing electron transfer. A linear correlation between the logarithm of the target concentration and the change in DPV current was observed in the range from 0.0 to 107 CFU/mL, with a limit of detection (LOD) of 3.0 CFU/mL. *E. coli* O157:H7, *P. aeruginosa*, *S. aureus*, *V. parahaemolyticus*, and their mixture were tested for selectivity. In comparison to the target, all of these bacteria demonstrated no significant DPV responses. Regarding reproducibility, the results obtained were considered acceptable in terms of relative standard deviation (RSD) at 5.65%. Sensor stability was evaluated, but the storage conditions have not been specified. The authors reported only the RSD value of 3.63%. Finally, the aptasensor was applied to spiked real samples of milk and orange juice, but recovery tests were not performed.

An immunosensor for *S. typhimurium* was developed based on a GCE modified with a nanocomposite including platinum nanoparticles (PtNPs) and Co/Zn-metal–organic framework, chitosan (CHI) and carboxylic multiwalled carbon nanotubes (PtNPs-Co/Zn-ZIF-8@C-MWCNTs/GCE) [103]. CHI, having a peculiar film-forming ability and biocompatibility, was used as a dispersing agent, while PtNPs were used as immobilizing sites for Abs by electrochemical deposition. The nanocomposite, including MOF and MWCNTs, increased the conductivity and stability of the sensing interface. In this case, a linear correlation between the logarithm of the target concentration and DPV current change of 1.3 × 10^2^ to 1.3 × 10^8^ CFU/mL was achieved, along with a LOD of 9.4. CFU/mL. *E. coli O157:H7*, *S. aureus*, *B. subtilis*, *L. casei*, and *C. perfringens* were evaluated as possible interferents; their responses were insignificant to that of the target. The repeatability of the immunosensor was investigated using five sensors and the same immunosensor five times consecutively. The RSD was 5.3% for independent measurements and 1.6% for consecutive measurements. The sensor stability was considered quite acceptable. In fact, after 15 days at 4 °C, the DPV response decreased by 13.30%. Finally, the sensor was applied to spiked real samples of pasteurized, skimmed and plain milk, with recoveries ranging from 94.07% to 105.76%.

Another immunosensor was assembled, immobilizing *Salmonella typhimurium* antibodies onto an ITO electrode modified with VSe_2_, a TMD, rarely used in the biosensing area [104]. A linear decrease in the DPV response with the log of increased pathogen concentrations from 10^1^ to 10^7^ CFU/mL, with a LOD of 0.0387 CFU/mL, was obtained. The signal decrease is probably due to the trapping of the target on the ITO surface, which blocks the electron transfer. *E. coli, S. aureus*, and *K. pneumoniae* were tested as possible interferents, and the results showed an interference of 12%, 5% and 2% for *S. aureus*, *E. coli* and *K. pneumoniae*, respectively. The repeatability of the immunosensor was investigated using the same immunosensor twenty times consecutively, but the authors did not report the RSD% value. Recovery tests were performed by applying the immunosensor to spiked real samples of sugarcane. The corresponding recoveries ranged from 96.2% to 99.0%.

An electrochemical MIP sensor for the detection of *Salmonella typhimurium* was developed using a SPAuE modified with PDA as the MIP. PDA was electrosynthesized on the electrode surface [105]. *S. typhimurium* was determined by CV. The current signal decreased with the increasing concentration of the target because the sensor has limited binding sites, and binding to higher concentrations of the target inhibits electron transfer. A linear range from 10 to 105 CFU/mL and a LOD of 10 CFU/mL were found. Bacteria similar in shape and size to, or even smaller than, *Salmonella typhimurium*, were identified as potential interferents, yet they did not significantly affect the target response. The applicability of the MIP sensor was analyzed, including spiked real samples of pork meat and milk, although the recovery tests were not conducted.

A simple immunosensor was prepared using a SPAuE modified with mercaptoacetic acid (MAA) as a suitable system to immobilize the antibody via a self-assembly monolayer (SAM) [106]. *S. typhimurium* was determined by EIS, and the decrease in impedance with increasing bacterial concentration could likely be explained by the particular interactions between the bacterial surface functionalities and the immobilized antibody, promoting electron transfer. A linear concentration range of 10–10^6^ cells/mL and a LOD of 10 CFU/mL were found. Long-term stability tests indicated that the immunosensor maintained over 90% of its initial response after 30 days at 4 °C. The authors claimed to have carried out tests to assess the sensor’s reproducibility and selectivity, but they have not given any additional information.

**Figure 8:**
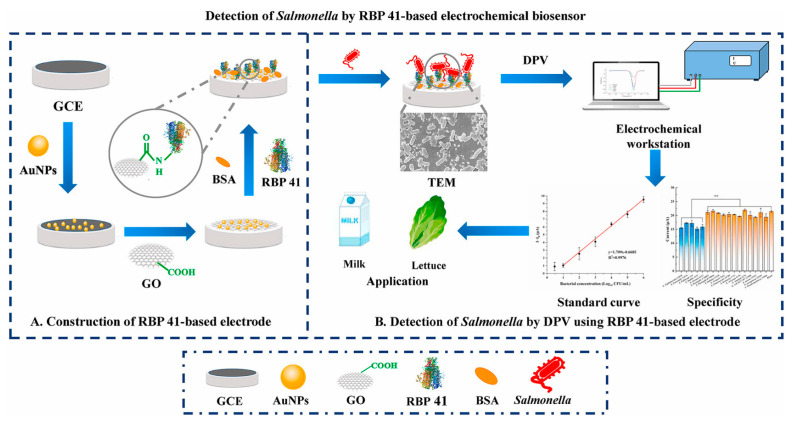
Scheme illustrating the biosensor assembling and the determination procedure of *Salmonella*. Reprinted with permission from [107]. Copyright 2025, Elsevier.

An electrochemical biosensor was developed using GCE modified with a layer-by-layer assembly of GO, AuNPs, and RBP 41. RBP 41 is an encoded phage receptor-binding protein (RBP) and acts as a biorecognition element for *Salmonella* [107] as illustrated in Figure 8.

It is well known that phages have been used as biorecognition elements, but they can cause the degradation of the bacterial target, undermining the sensor’s performance. On the other hand, phages interact with the bacteria through RBPs, which are specific, smaller, and resistant to sudden changes in temperature, pH, and protease activity. The biosensor was able to detect *Salmonella* in the concentration range from 3 to 10^6^ CFU/mL by using DPV, and the LOD was 0.2984 Log_10_ CFU/mL. Different types of *Salmonella* species were tested for the selectivity tests, including *S. Enteritidis*, *S. Pullorum*, *S. Dublin* and *S. Javiana*. The results indicated that their responses were quite similar to those of the control sample. In addition, the biosensor was applied to spiked samples of milk and lettuce, and the recoveries were in the range from 84.0% to 120.0%.

Another electrochemical genosensor based on CRISPR/Cas12a was developed for the detection of *Salmonella Typhimurium* [108]. CRISPR/Cas12a technology can recognize specific sequences of target DNA, as indicated in the previous examples [86,96]. Colloidal gold (alias AuNPs) and MXene (CG@MXene) nanocomposite were immobilized on GCE via electrostatic adsorption, enhancing the analytical performance of the genosensor by reducing background noise. A linear decrease in the DPV response with the increase in pathogen concentrations from 10^2^ to 10^7^ CFU/mL, with a LOD of 160 CFU/mL was obtained. Different pathogens were used for the specificity tests, such as *Escherichia coli*, *Staphylococcus aureus*, *Bacillus cereus*, and *Shigella flexneri*. The results indicated an acceptable specificity with a cross-reactivity ranging from 5.70% to 19.19%. Long-term stability was investigated, and after 7 days at 4 °C, a decrease in the DPV signal of only 10% was observed. Recovery tests were performed using spiked samples of chicken meat, and the yielding recoveries ranged from 100.46% to 106.37%.

As the last example, we introduce an immunosensor based on AuE modified with a composite containing Fe_3_O_4_ nanoparticles and an ionic liquid (1-butyl-3-methylimidazolium hexafluorophosphate) [109]. The antibody was immobilized on the composite after the deposition of a poly(glutamic acid) (PGA) layer. PGA is stable, and its free carboxyl groups are appropriate for binding an antibody. Fe_3_O_4_ nanoparticles have large specific surface areas and are biocompatible, while IL shows high ionic conductivity and good stability. The DPV response decreased linearly with increasing pathogen amount in the range of 3.65 × 10^2^–3.65 × 10^8^ CFU/mL, probably because the steric hindrance on the electrode surface increased, limiting the electron transfer. A LOD of 1.12 × 10^2^ CFU/mL. *Listeria monocytogenes*, *Shigella sonnei*, *Proteus bacillus vulgaris*, *Escherichia coli O157:H7*, *Pseudomonas aeruginosa*, and *Staphylococcus aureus* were included in the specificity tests, and the DPV response of *Salmonella typhimurium* was higher than those of the other pathogens. The reproducibility was satisfactory in terms of RSD (2.80%). Concerning the long-term stability trials, after 15 days at 4 °C in the refrigerator, a decrease in DPV signal was 15.4%. Finally, the applicability of the immunosensor was analyzed using spiked real milk samples, with recoveries ranging from 99.40% to 110.13%.

#### 3.2.4. *Escherichia coli*

*Escherichia coli* is a Gram-negative, bacilliform bacterium that belongs to the same family as *Salmonella* [110,111]. Some varieties of *E. coli* are harmless and are normally found in the human intestinal bacterial flora. However, other strains of *E. coli* can cause intestinal infections that may be severe and potentially life threatening. These diseases can be transmitted through food or drink contaminated with these bacterial strains.

We want to report as a first example of an electrochemical biosensor for determining *E. coli,* a label-free electrochemical biosensor using phage EP01 [112]. The biosensor was assembled by incorporating phages conjugated carboxyl graphene oxide (CFGO) and conductive carbon black (CB) onto GCE. The combination and synergistic action of EP01, CFGO, with its carboxyl groups and CBs, guarantees the sensor’s high specificity, electron conductivity, easy functionalization and large surface area. *E. coli* was determined by EIS, and a linear concentration range of 10^2^–10^7^ CFU/mL and a LOD of 11.8 CFU/mL were obtained. *Klebsiella pneumoniae*, *Pseudomonas aeruginosa*, and *Salmonella* were tested to verify the selectivity. Their responses were comparable to the EIS response of the blank control. The long-term stability was also investigated, and after 7 days at 4 °C, the electrochemical response decreased by only 1.57%. The reproducibility was satisfactory with an RSD of 2.7%. The biosensor was employed to analyze fresh milk and raw pork samples. The recovery values ranged from 60.8% to 114.2%.

The next example introduces an electrochemical biosensor based on the CRISPR/Cas12 system, nucleic acid aptamer, triple helix DNA and entropy-driven amplification reaction (EDC) [113], assuring high precision and accuracy in the detection of *E. coli* [114]. A triple-helical DNA was used as a recognition element, substituting antibodies with nucleic acid aptamers. *E. coli* was determined by SWV in the linearity range of 1 × 10^2^–1 × 10^7^ CFU/mL with a LOD of 5.02 CFU/mL. To verify the specificity of the biosensor, different species of bacteria were used to test the sensor’s specificity, including *Salmonella* and *Staphylococcus aureus* and the corresponding responses were similar to those of the blank control. Pure water and pure milk spiked samples were considered for investigating the applicability of the sensor, with recoveries ranging from 95.76% to 101.20%.

An electrochemical sensor based on an antimicrobial peptide (AMP) and a hydrophilic tannic acid (TA)-polyethyleneimine (PEI) hydrogel deposited on a gold electrode (Au/TA-PEI/AMP 3) was prepared for the detection of *E. coli* [115]. Three AMPs were obtained from magainin 1 and clavanin A through peptide engineering. Subsequently, one of these, indicated as AMP 3, evidenced the highest antimicrobial activity against *E. coli*. Consequently, it was selected as a bioreceptor. The 3D network structure of TA-PEI/AuE improved the electron transfer from and to the sensing interface. DPV was employed as an electrochemical technique, and the LOD of the biosensor was 3.4 CFU/mL, with a linear detection range of 10^1^–10^5^ CFU/mL. Gram-positive bacteria (*L. monocytogenes* and *S. succinus*) and six different Gram-negative bacteria, including *V. parahaemolyticus*, were used for testing the sensor specificity. The results indicated that none of them significantly affected the DPV response of *E. coli.* The reproducibility was acceptable with an RSD of 5.17%. The long-term stability was addressed: the authors indicated that after 10 days at 4 °C, its response remained almost stable. Spiked milk samples were used to verify the applicability of the sensor, with recoveries ranging from 89.1% to 122.0%.

An electrochemical genosensor was developed for determining *E. coli,* using trigging isothermal amplification (TICA) and a magnetic nanocomposite including Fe_3_O_4_ nanoparticles, AuNPs, and COF, deposited on an Au electrode [116]. The synergistic action of the nanomaterials and COF guaranteed a high surface area and improved the electron transfer, while TICA enhanced the sensitivity of the sensing system. The pathogen was determined by CV, and a linearity range of 10^2^–10^9^ CFU/mL with a LOD of 10 CFU/mL was found. Investigating the long-term, the sensor was kept at 4 °C for one month, and the electrochemical signal only decreased by 6.28%. The reproducibility results were considered acceptable with an RSD of 4.38%. *P. aeruginosa*, *S. aureus*, *S. pyogenes*, *S. enteritidis*, *S. pneumoniae* and *S. enteritidis* were used to verify the specificity of the genosensor, and the results indicated that none of them significantly affected the genosensor response. Finally, the applicability to spiked real samples of orange juice was analyzed, obtaining recoveries from 92.0% to 109.0%.

**Figure 9:**
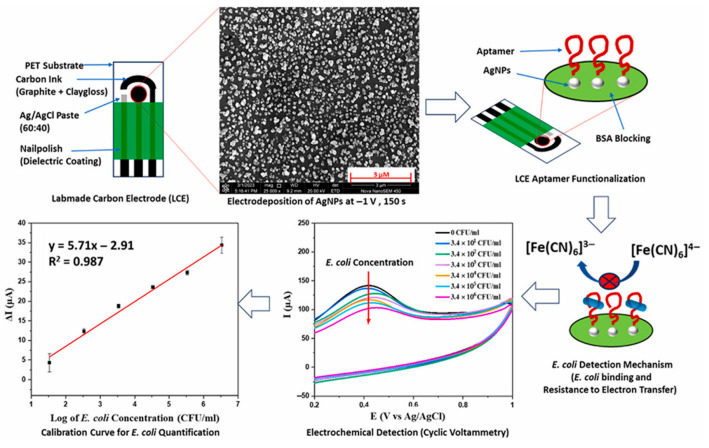
Scheme illustrating the aptasensor development and detection mechanism of *E. coli.* Reprinted with permission from [117]. Copyright 2025, Elsevier.

A homemade SPCE was used for developing an aptasensor for *E. coli*, including electrodeposited AgNPs and an aptamer immobilized via drop casting [117], as shown in Figure 9.

*E. coli* was analyzed via CV, and a linear concentration range of 3.4–3.4 × 10^6^ CFU/mL and a LOD of 3.4 CFU/mL were achieved. *E. casseliflavus*, *S. marcescens*, *B. subtilis*, *B. cereus*, and *S. aureus* were tested for the aptasensor selectivity. The results evidenced negligible interference from the bacteria investigated. The reproducibility results were considered good with an RSD% of 1.71%. The aptasensor was stored at 4 °C for four weeks, and a decrease of only 1.15% was observed. The sensor was applied to spiked milk and water samples, but the recovery tests were not performed.

A multi-analyte electrochemical aptasensor for detecting *Escherichia coli*, *Staphylococcus aureus*, and *Salmonella typhimurium* was realized using a SPCE modified with two-dimensional carboxyl Ti_3_C_2_T_x_ (2D C-Ti_3_C_2_T_x_) nanosheets as a signal amplifier and sensing platform, along with two-dimensional Zn-MOF (2D Zn-MOF) as a signal probe [118]. The aptamers were immobilized on C-Ti_3_C_2_T_x_ through an amidation reaction. The target bacteria were captured by the aptamer, resulting in a decrease in the peak current of 2D Zn-MOF measured using DPV. This decrease correlates with an increase in the concentration of the pathogens. Different pathogenic bacteria can be analyzed by simply replacing the aptamer. The detection limits for *E. coli*, *S. aureus*, and *S. typhimurium* are 6, 5, and 5 CFU/mL, while the linearity range is from 10 to 10^6^ CFU/mL for all the bacteria examined. Different bacteria were used for the specificity tests, and almost no change in DPV current was evidenced. Reproducibility and stability tests were performed for *E. coli*. The reproducibility was satisfactory with an RSD of 1.49%. Concerning the stability, the DPV signal remained almost unchanged, but the storage conditions were not specified. The aptasensor was applied to spiked samples of egg and milk. The corresponding recoveries of *E. coli*, *S. aureus*, and *S. typhimurium* were 82.5–132%, 74.7–134%, and 93.3–138%, respectively.

We would like to comment briefly on the biosensor examples for foodborne bacteria determination. First, there is no preferred format, and we can find an equal number of examples of aptasensors, immunosensors, and genosensors. At the same time, we have discovered a significant number of biosensors based on bacteriophages. Phages, in comparison with other biorecognition elements such as aptamers or antibodies, are certainly less expensive, can be easily functionalized and are quite stable regardless of the experimental conditions [119,120]. Almost all the biosensors reported have been used on real food samples, but often they are not validated using conventional analysis methods. Moreover, reproducibility or stability data are not always included, while selectivity data tend to be emphasized.

Analytical performances of the electrochemical sensors for bacteria determination, together with corresponding sensor formats, are summarized in Table 2.

### 3.3. Pesticides

Pesticides are chemical compounds widely used in agriculture to eliminate the effects of bacteria, viruses or pests on plant growth, limit or prevent the growth of weeds and combat disease-carrying insects [121]. Such a massive use of pesticides entails risks and side effects, given that only a small part of the amount of pesticides used acts effectively against bacteria, viruses, weeds or insects, the rest is unfortunately dispersed in the environment. Consequently, an accurate and rapid analysis of pesticides is necessary because of their environmental impacts and associated risks to human and animal health. According to the target, pesticides can be defined as insecticides, fungicides, herbicides, etc. In addition, the main classes of pesticides are the following: carbamates, organophosphates, and pyrethroids, among others.

Glyphosate, *N*-(phosphonomethyl)glycine (GLP), is a broad-spectrum, non-selective herbicide and is used to control weeds after they have already emerged from the soil. On the other hand, GLP is associated with different pathological effects and diseases, especially endocrine dysfunctions and cancer [122].

As a first example, we present an electrochemical sensor for determining GLP based on copper-organic framework (MOF) integrated with a carbon nanofiber matrix. The Cu-organic framework was synthesized starting from 3,5-pyrazoledicarboxylic acid (PZDA) as the organic ligand and via a one-pot solvothermal approach. Cu-PZDA was incorporated during the synthetic procedure in carbon nanofibers (Cu-PZDA/CNFs) [123]. Cu-PZDA/CNFs composite was deposited on GCE, and the modified electrode was used for determining GLP. Cu-PZDA combined its properties, such as thermal stability and large surface area, with the good conductivity, the delocalized π-electronic system and a higher specific surface area of CNFs. GLP was determined by DPV in the range of 0.01–200 μM with a LOD of 3.12 μM. Different types of pesticides, such as fenitrothion, chlorpyrifos, malathion, dichlorvos, acetamiprid and carbendazim, were used for the selectivity tests, and none of them significantly affected the GLP DPV response. The sensor was stored in the freezer for 45 days, and the electrochemical response decreased only by 2.24%. The reproducibility was satisfactory in terms of RSD (1.34%). Finally, Cu-PZDA/CNFs/GCE was applied to actual samples of beetroot juice and lettuce extracts. The corresponding recoveries ranged from 98.01% to 101.83% and were comparable with those obtained with HPLC.

Pyrethroid pesticides mimic hormones and can cause damage to the endocrine system in some cases, potentially interfering with hormone signaling mechanisms [124]. Identifying and determining residues of these insecticides in food is essential due to their widespread agricultural use. Deltamethrin is a pyrethroid insecticide classified as a cyclopropane carboxylate ester. It plays a key role in controlling malaria vectors such as mosquitoes and bed bugs. Deltamethrin is toxic for fish, while at high doses it may be neurotoxic for humans. Pyrethroids like deltamethrin are considered allergens, inducing asthmatic crisis in predisposed subjects.

An electrochemical sensor was developed using an MIP to detect deltamethrin [124]. The sensor was assembled by electrodeposition of Co_3_O_4_ electrochromic film on ITO, followed by electropolymerization of o-phenylenediamine (o-PDA) with deltamethrin as a template molecule. This electrochromic pesticide sensor is assumed as an electrochemical-MIP sensor including an electrochromic film. Quantitative and qualitative pesticide determination is available with this system, which is more affordable than a common MIP electrochemical sensor. It is possible to determine the target pesticide and identify it through the color variations, depending on the different concentrations of the analyte. The insecticide was determined by CV in the range of 2.82–56.5 nM with a detection limit of 1.53 nM. Parathion and acetamiprid were selected to evaluate the selectivity of MIP/Co_3_O_4_/ITO, and the deltamethrin response was not affected by the presence of the other pesticides. The sensor reproducibility was good in terms of RSD (1.78%). Tomato, salad, grapefruit, and orange samples were analyzed by MIP/Co_3_O_4_/ITO, and the results were validated with HPLC. The recovery values ranged from 97.20% to 105.33%.

Thiabendazole (TBZ) is a fungicide and insecticide, commonly utilized in agriculture to eliminate the presence of downy mildew and infestation on crops [125]. Residues of this insecticide have been identified in agricultural products, and it is demonstrated that TBZ can induce damage to the thyroid and to the liver, and it has been associated with some severe cancers.

We introduce an electrochemical sensor based on a GCE modified with MoS_2_ nanosheets deposited on silver nanowires (Ag NWs@MoS_2_) [125]. The combination of electrical conductivity, large surface area, and adsorption capabilities of MoS_2_, a two-dimensional TMD, with the conductivity, surface area and electrocatalytic properties of AgNWs enhanced the analytical performances of electrochemical sensors. TBZ was determined by SWV, and the sensor evidenced a linear range of 0.05–10 μM with a limit of detection of 1.75 nM. The repeatability was investigated involving six repetitive measurements at a single electrode, and the corresponding RSD was 1.72%. On the other hand, the reproducibility was addressed using six sensors prepared simultaneously, and the corresponding RSD% was 5.23%. Repeatability and reproducibility were considered satisfactory. Ascorbic acid (AA), citric acid (CA), glucose, and acetylthiocholine chloride (ATCL) were tested as possible interfering compounds, and none of them significantly affected the TBZ response. Pear and apple samples were used to evaluate the applicability of the sensor, and the results were validated with HPLC. The corresponding recoveries were in the range of 95.5–103.6%.

Dinotefuran (DNF, (*RS*)-1-methyl-2-nitro-3-(tetrahydro-3-furylmethyl)guanidine) is a neonicotinoid insecticide, and its antagonistic action on the nicotinic acetylcholine receptors of insects has a neurotoxic effect. DNF residues in water, soils, grains, fruits, and vegetables can be present in the food chain and lead to serious health issues for consumers.

A flexible electrochemical sensor based on poly(caprolactone)/polypyrrole/β-cyclodextrin (PCL/PPy/β-CD) composite was prepared for determining DNF [126]. Conductive PPy was chemically polymerized on the biodegradable PCL fibers, prepared by the electrospinning process. β-CD was electropolymerized in the void of conductive porous PCL/PPy membrane, so a secondary pore framework was obtained. PPy could enhance the electron transfer from and to the electrode, and β-CD could recognize the target molecule. DNF was analyzed by DPV and two linear concentration ranges were observed: the first of 0.2–5 μM and the second of 5–50 μM with a LOD of 0.05 μM. Glucose and saccharose were used as interfering biological molecules, and their influence on DNF response was less than 5%. Six PCL/PPy/β-CD electrodes were employed to test the sensor reproducibility, and the results were acceptable (RSD% 4.76). The operational stability was evaluated by measuring the same electrode 10 times in the presence of DNF, and after six measurements, the signal decreased by only 4.41%. The sensor was then applied to spiked real samples of rice with a recovery range of 96.67–103.65%.

Carbendazim (CBZ, methyl benzimidazol-2-yl carbamate) is a broad-spectrum fungicide to combat and treat several diseases in crops. Nonetheless, its effects on human health can be associated with liver diseases, chromosomal abnormalities and cancer [127].

A GCE was modified with Co(OH)_2_/TiO_2_ to develop an electrochemical sensor for determining CBZ [127]. TiO_2_ is biocompatible, stable, non-toxic and cost-effective with peculiar adsorption properties, while Co(OH)_2_ is a good electrocatalyst. The synergistic combination between Co(OH)_2_ and TiO_2_ improved the electrode conductivity and electron transfer capability. CBZ was analyzed by CV and two linear concentration ranges were obtained: the first of 0.039–0.399 μM and the second of 0.399–2630.1 μM, with a LOD of 0.007 μM.

Carbofuran, flutamide, D-glucose, methyl parathion, hydroquinone, diuron, ascorbic acid, and dopamine were considered as possible interfering molecules, and the CBZ response was not affected by their presence. Four sensors were used for the reproducibility tests, yielding an RSD value of 1.18%, while five consecutive analyses using the same sensor were considered for the repeatability, obtaining an RSD value of 1.12%. Apple, orange, as well as vegetables such as cabbage, carrot, and tomato, were considered for evaluating the sensor applicability to real samples, with recoveries ranging from 96.84% to 106.69%.

Diethofencarb (DFC, propan-2-yl *N*-(3,4-diethoxyphenyl)carbamate) belongs to the *N*-phenyl carbamate chemical family [128]. DFC is widely utilized as a fungicide in agriculture, particularly on tomatoes, grapes, peaches, and plums. However, excessive and uncontrolled application of DFC may adversely affect the environment, as well as human and animal health.

**Figure 10:**
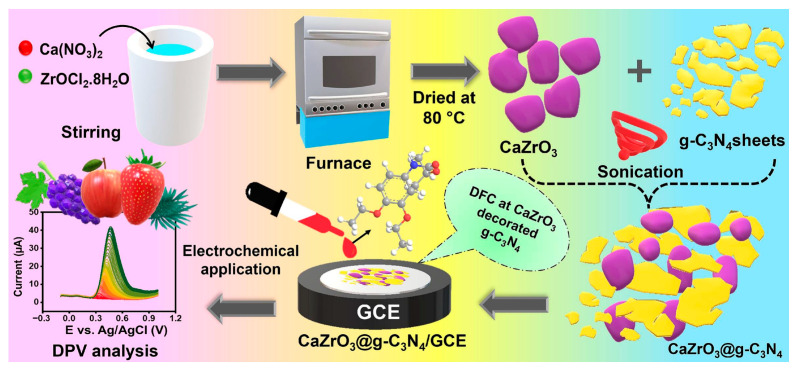
Scheme illustrating the composite synthesis, the sensor development and the detection approach of DFC. Reprinted with permission from [128]. Copyright 2025, Elsevier.

An electrochemical sensor was assembled using CaZrO_3_ incorporated into g-C_3_N_4_ nanosheets [128] as shown in Figure 10.

Calcium zirconate (CaZrO3) belongs to the family of perovskite oxides, recognized for their role in several electrochemical devices and applications due to their chemical stability, corrosion resistance, and structural flexibility. Generally, these compounds exhibit lower conductivity at room temperature; to tackle this issue, they can be combined with more conductive materials. DFC was quantified by DPV, achieving a linearity range of 0.01–230.04 μM and a LOD of 1.8 nM. Dopamine (DA), carbofuran (CBF), diuron (DU), promethazine (PM), isophorone diamine (IPD), p-cysteine desulfhydrase (PCD), carbendazim (CBZ), ascorbic acid (AA), Glucose (GLU), uric acid (UA), urea, and l-cysteine desulfhydrase (LCD) were indicated as interfering molecules, and their presence affected less than 5% of the DFC response. Reproducibility and repeatability were acceptable, yielding the RSD values of 3.12% and 2.36%, respectively. Finally, the operational stability was verified, and after 20 consecutive measurements, the response decreased by only 4.29%. Strawberry, grapes, spinach, and apple spiked samples were analyzed to investigate the sensor applicability, and the recoveries ranged from 98.20% to 99.80%.

#### Organophosphate Pesticides (OPs)

Organophosphate pesticides (OPs) are among the most commonly used pesticides because they are both effective and affordable. They are cholinesterase inhibitors and degrade easily in the sun, air, or soil, but traces can still be found in food and in the drinking water [129]. Consequently, they can affect the metabolic processes because they can penetrate the mammalian tissues. Finally, we can underline that the exposure to OPs can induce different levels of toxicity in humans, animals, plants, and insects. OPs are chemically produced by the reaction of alcohol with phosphoric acid, and their structure contains a phosphoryl group, two lipophilic groups bonded to the phosphorus, and a leaving group, usually a halide. The most common OPS are chlorpyrifos, dichlorvos, malathion, paraoxon, parathion, and phoxim.

As a first example, we report an electrochemical biosensor for the detection of chlorpyrifos (CFP), using a GCE modified with MXene nanosheets, AuPt bimetallic nanoparticles (AuPtNPs), chitosan (CHI) and the enzyme acetylcholinesterase (AChE) [130]. The combination of MXene and AuPtNPs improved the adsorption capacity, stability, and electron transfer capability of the sensing interface and supported the signal amplification. CHI facilitated the enzyme immobilization on the MXene/AuPtNPs nanocomposite. The electroanalytical determination is based on the AChE inhibition in the presence of acetylthiocholine (ATCl) by CFP. CFP was considered an inhibitor of AChE because it prevents the hydrolysis of acetylthiocholine, producing thiocholine (TCh). It was measured by DPV, and the enzymatic inhibition resulted in a decrease in TCh signal when the chlorpyrifos concentration increased. A linear relationship was obtained in the range of 10^−8^–10^−3^ mg/mL, and the LOD was 1.55 pg/mL. The repeatability and stability of the biosensor were studied and were acceptable, yielding the RSD values of 3.9% and 5.1%, respectively. The long-term stability was investigated, and after 30 days at 4 °C, the DPV signal decreased by 17%. Glucose, urea, citric acid, and AA were selected as possible interferences, but they did not interfere significantly. Apple and cabbage spiked samples were tested to evaluate the biosensor applicability, and the following recovery range was found: 95.44–102.81%.

Another biosensor for the determination of CFP was prepared using a SPCE modified with copper nanowires (CuNWs), reduced graphene oxide (rGO) and the enzyme AChE, involving, as in the previous example, the enzymatic inhibition [131]. The biocompatible CuNWs/rGO nanocomposite amplified the electrochemical signal and decreased the oxidation potential of the OP. In addition, it improved the conductivity and the electron transfer of the sensing platform and supported the immobilization of AChE. CFP was determined by CV and a linear concentration range from 10 μg/L to 200 μg/L, with a limit of detection of 3.1 μg/L was found. The biosensor was not significantly affected by the presence of metal ions in determining OP. Drinking water and orange juice samples were evaluated for investigating the biosensor applicability, with recoveries ranging from 96.67% to 105.65%.

As the last example of a chlorpyrifos biosensor, we report an electrochemical acetylcholinesterase (AChE) sensing platform [132]. The enzyme was immobilized in a CHIT film deposited on GCE, modified with a lamellar Ti_3_C_2_ MXene (TM) decorated with Pt-doped MoS_2_ nanosheets (Pt/MoS_2_/TM). Pt/MoS_2_/TM nanocomposite showed a peculiar two-dimensional nanosheet/laminar hybrid structure and was biocompatible, with high electrical conductivity, stability and a large specific surface area. CFP was determined by DPV, using the AChE inhibition, and the corresponding linear concentration range was 10^−6^–1 μM with a LOD of 4.71 × 10^−13^ M. The repeatability was tested using ten measurements, at the same biosensor, with an RSD of 1.9%. The reproducibility was investigated using five biosensors with an RSD of 1.25%. Finally, the storage stability was analyzed, and after seven days at 4 °C, the DPV response decreased by 9.62%. Glucose, urea and some metal ions were considered as interferences. The results showed that there was no significant change in the current response value in their presence. Strawberries, pakchoi and Chinese chive are chosen as the test samples for investigating the real applicability of the biosensor, and the corresponding recoveries ranged from 94.81% to 104.00%

In the next example, an acetylcholinesterase (AChE) biosensor including titanium oxide nanorods (TiO_2_-NRs) and reduced graphene oxide (rGO) for detecting dichlorvos (DDVP), another widely used OP, was described [133]. A GCE was modified by a layer-by-layer assembly procedure to sequentially deposit a chitosan (CHI)-modified reduced graphene oxide (CHI@rGO) nanocomposite, gold Au-NPs, TiO_2_-NRs, and AChE. rGO increased the biosensor’s specific surface area and enhanced the electrochemical response. CHI improved the mechanical strength of rGO and the sensor’s stability, while AuNPs and the biocompatible TiO_2_-NRs enhanced conductivity and the electron transfer rate to and from the electrode surface. Furthermore, TiO_2_-NRs provided a three-dimensional structure to the biosensing interface. DDVP was determined by DPV, involving the inhibition of AChE and two linear ranges from 2.26 nM to 56.5 nM and from 56.5 nM to 565 nM were found, with a LOD of 2.23 nM (0.483 ppb). Glucose and bovine serum albumin (BSA), together with some metal ions were tested as possible interferences, and the analytes evidenced no effect on DDVP analysis. The storage stability was investigated at room temperature, and after 23 days, the signal decreased by less than 10%. Cabbage and orange juice samples were used to test the applicability of the biosensor, and recoveries ranging from 90.3% to 101.6% were obtained.

**Figure 11:**
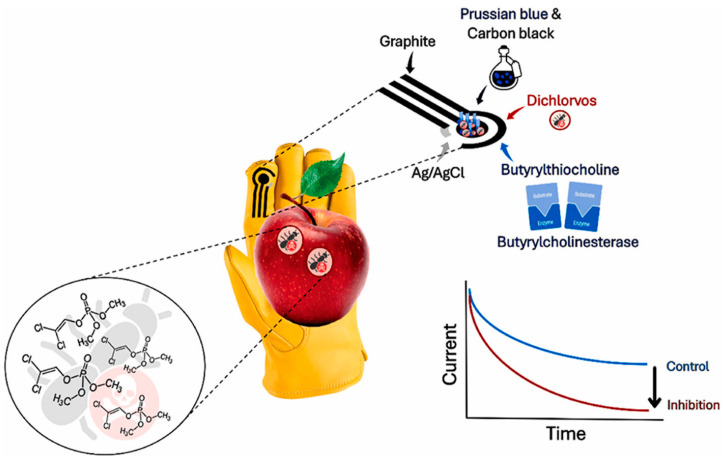
Scheme illustrating the composite synthesis, the sensor development and the detection approach of DDVP. Reprinted with permission from [134]. Copyright 2025, Elsevier.

A hybrid composite including Prussian blue nanoparticles, carbon black (CBs) and butyrylcholinesterase enzyme (BChE) was deposited onto a screen-printed electrode, developed on a glove for detecting DDVP [134] as illustrated in Figure 11.

DDVP was quantified by chronoamperometry, using the inhibition of BChE and a linear concentration range from 0.0 up to 20 ppb and a LOD of 1 nM (0.3 ppb) were achieved. The repeatability, as the relative standard deviation, was calculated on four measurements, obtaining an RSD value of 7%. The selectivity was evaluated in the presence of salts and substances potentially toxic for the enzyme, such as potassium nitrate, ammonium sulfate, mercury, copper and cadmium, and the percentage of inhibition in their presence was negligible compared to that obtained with the same concentration of DDVP. Apple and orange peel samples were used to investigate the applicability of the “biosensing” glove. First, the biosensing platform was used in solution and in a “dry” mode. Recoveries were calculated using two concentrations of DDDVP (10 and 50 ppb) and resulted equal to 81–104% and 91–115%, for orange tested through solution and dry modes, respectively and equal to 83–95% and 106–97% for apple tested through solution and dry modes, respectively.

Malathion (MAL) and carbendazim (CBZ) are two organophosphate pesticides and fungicides and an electrochemical sensor based on a graphite-epoxy composite electrode (GECE) was assembled for their simultaneous determination [135]. GECE was modified with GQDs, covered with a MIP. MAL and CBZ were employed as the template molecules, ethylene glycol dimethyl acrylate as the cross-linking agent, and methacrylic acid as the functional monomer. GQDs@MIP (MAL) and GQDs@MIP (CBZ) were then incorporated into the graphite-epoxy composite electrode. MAL and CBZ were quantified by DPV simultaneously. A linear concentration range of 0.01–55.00 μM for MAL and of 0.05 to 45.00 μM for CBZ was found. The LOD was 2 nM for MAL and 1 nM for CBZ. After two months under undefined experimental conditions, the DPV responses of MAL and CBZ retained 89.2% and 89.7% of their initial values. Several pesticides such as deltamethrin (DLM), fenitrothion (FNT), diazinon (DZN), and CFP were selected as possible interfering compounds. Considering the experimental results, these pesticides did not constitute a problem for the MAL and CBZ detection. Cucumber, tomato and grape juice samples were analyzed to study the sensors’ applicability, yielding recoveries in the range of 97.75–109.6%.

A non-enzymatic electrochemical sensor of MAL was realized based on a GCE modified with g-C_3_N_4_, CuO and biochar (B) [136]. g-C_3_N_4_ has tunable surface chemistry, and the composite of g-C_3_N_4_ with copper oxide (CuO) exhibits high conductivity and chemical stability. Moreover, the inclusion of biochar, produced from biomass, with well-known absorption properties and a large surface area, provides active sites for improving the sensor’s electrochemical performance. MAL was determined by square-wave anodic stripping voltammetry technique (SWASV) with a linearity range of 0.18–5.66 pg/mL and a LOD of 1.2 pg/mL. The repeatability and reproducibility were acceptable, and the corresponding RSD values were 7.02% and 9.69%, respectively. Methyl parathion (MP), CFP, and different metal ions were identified as possible interferences. The greatest interference was evidenced by high concentrations of Ni^2+^, Al^3+^, K^+^, and Fe^3+^, suggesting the detection of MAL in samples with lower concentrations of these metal ions. On the other hand, the other pesticides did not significantly affect the detection of MAL. The sensor was finally applied to spiked real samples of tomato and apple extracts with a recovery percentage of 87.64–120.59%.

A two-dimensional Ti_3_C_2_T_x_/MWCNT-OH nanocomposite, casted on a GCE, was utilized to assemble an electrochemical sensor for detecting ethyl-paraoxon [137]. It is well known that Ti_3_C_2_T_x_ exhibits metallic conductivity, proper hydrophilicity, fast electron transfer rates, and a large surface area for interaction with target molecules. The combination of MWCNT-OH, obtained through acid treatment of MWCNTs, enhances the conductivity and mechanical strength of the sensing layer. The acid functionalization of MWCNTs supports their inclusion into the 2D structure of the MXene. A linear concentration range from 0.1 to 100 μM and a LOD of 10 nM were achieved using DPV. Diazinon, carbaryl, AA, and glucose were considered as possible interfering molecules, but they exhibited negligible or minimal effects on the detection of the pesticide. The reproducibility and stability were evaluated. The reproducibility was investigated using six different electrodes, yielding an RSD of 4.2%. The stability was evaluated by testing the same electrode after a storage of five days, under unspecified experimental conditions and no data concerning the DPV response was reported. Spiked samples of green and red grapes were employed to verify the applicability of the sensor, but the recovery tests were not performed.

As the next example, we present an electrochemical biosensor for the determination of MP, based on the inhibition of AChE, using a GCE modified with a zinc oxide-reduced graphene oxide (ZnO-rGO) composite [138]. Graphene and its derivatives, such as rGO, show a thin layer structure, a large specific surface area, fast electron transfer, biocompatibility, and affinity for biomolecules or enzymes. The integration with metal oxides into the graphene structure improves the conductivity and the specific surface area, introducing more catalytically active sites and accelerates the electron transfer from and to the sensing interface [138]. MP was detected via DPV, and a linearity range of 0.01~1000 ng/mL with a LOD of 0.00463 ng/mL was obtained. Glucose, citric acid, sucrose, and different metal ions were included for testing the selectivity of the biosensor, but negligible or minimal interferences were evidenced. The reproducibility was acceptable, obtaining an RSD value of 2.75%. The storage stability was addressed, and after 15 days at 4 °C, the DPV signal decreased by only 3.7%. Spiked samples of cucumber and apple were analyzed to study the applicability of the sensor, and the recoveries were in the range from 90.92% to 108.4%.

As the last example, we introduce a biosensor for the analysis of phoxim, using a GCE modified with Ti_3_C_2_ MXene, a TMD like MoS_2_, AuNPs and AChE [139]. The two-dimensional and multi-layer framework of MXene enables TMD to be included efficiently, increasing the specific surface area. Additionally, the combination of AuNPs with MoS_2_ further improves synergistically the conductivity, while the multilayer composite system supports the proper immobilization of AChE. The biosensor showed a linear response to phoxim in the range of 1 × 10^−13^–1 × 10^−7^ M with a LOD of 5.29 × 10^−15^ M. The repeatability and the reproducibility were addressed, obtaining satisfactory results in terms of RSD values, 2.3% and 2.8%, respectively. The long-term stability tests indicated that after four weeks of storage at 4 °C in the refrigerator, the biosensor maintained 84.3% of its initial DPV response Glucose, citric acid, aspartic acid, serine, arginine and some metal ions were included for testing the selectivity of the biosensor, but negligible or minimal interferences were evidenced. Spiked samples of winter jujube, red date, raisin, and apricot were tested to investigate the possible applicability of the biosensor. The recoveries were in the range of 99–107%, and the results were validated with HPLC.

We would like to add some consideration to the examples reported in this section. It should be noted that biosensors based on enzymatic inhibition are present in a large number, but they are no longer the preferred sensor format. The development of nanomaterials, the design of nanocomposites and the introduction of molecularly imprinted polymers have probably contributed to the growing use of non-enzymatic sensors. It is important to emphasize that the applicability of various sensors to real samples is always assessed, but validation with conventional analysis systems is often missing. Analytical performances of the electrochemical sensors for pesticide determination, together with corresponding sensor formats, are summarized in Table 3.

### 3.4. Antibiotics

Antibiotics are chemical compounds of natural or synthetic origin with antibacterial and antimicrobial properties. For this reason, they have been widely used to treat diseases in humans and animals. They are also used as preservatives and additives in cases where refrigerators or refrigeration systems cannot be used. To conclude, it is worth noting that animal husbandry and aquaculture employ a significant amount of antibiotics. Excessive and uncontrolled use of antibiotics results in their accumulation in milk or food products, such as meat or farmed fish. Consuming contaminated food or milk can lead to resistance to antibiotics or allergic reactions in humans, which are both undesirable and dangerous for human health [140,141,142]. It is, therefore, necessary to develop methods that are fast, sensitive, selective, low cost, and easy to use for detecting antibiotics in food.

Tobramycin is widely used as a broad-spectrum antibiotic in livestock due to its low cost and water solubility [142]. An aptasensor for detecting tobramycin was prepared using exonuclease III (Exo III) and metal ion-dependent DNAzyme recycling and hybridization chain reaction (HCR) signal amplification cascades. The analyte links with the aptamer-containing hairpin probe inducing its conformation to expose the foothold sequence. Consequently, Exo III-based catalytic digestion of the secondary hairpin was activated, producing many DNAzyme strands. The hairpins immobilized on AuE were cut by the DNAzymes, and ssDNAs were created. ssDNAs triggered the HCR formation of many long MB-tagged dsDNA polymers on the electrode. Next, the oxidation of the MB labels produced SWV responses for sensing tobramycin within the 5–1000 nM concentration range, with a LOD of 3.51 nM. The reproducibility and the repeatability were considered and the corresponding RSD values were satisfactory: 6.3% and 2.7%, respectively. The stability was evaluated by storing the aptasensor at 4 °C for 14 days, and a decrease in SWV response of 8.2% was observed. Different antibiotics were tested to investigate the selectivity of the aptasensor. Penicillin, sarafloxacin and streptomycin evidenced electrochemical responses lower than those of the blank sample and tobramycin. Gentamicin, kanamycin and ampicillin showed responses slightly higher than that of the blank, but much lower than that of tobramycin. Finally, the aptasensor was applied to spiked samples of milk and the recoveries ranged from 97.6% to 102.7%.

Monensin (MON) is a natural antibiotic synthesized by *Streptomyces cinnamonensis* and used in animal husbandry [143]. An electrochemical immunosensor for the determination of MON was developed using anti-MON monoclonal antibodies (anti-MON mAbs) and a nanocomposite (Ce-MOF@AgAuNPs). The nanocomposite included silver and gold bimetallic nanoparticles (AgAuNPs) incorporated in a cerium-based metal–organic framework (Ce-MOF). Ce-MOF acted as a dispersing agent for AgAuNPs. On the other hand, AgAuNPs increased the conductivity and provided a proper microenvironment for the immobilization of anti-MON mAbs. MON was detected by DPV, and a linearity range of 0.05–250 ng/mL with a LOD of 0.008 ng/mL was found. Maduramicin, lasalocid, sulfamethazine, sulfadiazine, flavomycin and olaquindox were tested to verify the selectivity of the immunosensor. The electrochemical signal of the various interfering antibiotics did not differ significantly either in the presence or absence of MON and was comparable to the sample blank. The reproducibility was tested: the RSD value of intra-electrode reproducibility was 1.68%, while that of the inter-electrode reproducibility was 0.96%. Finally, the stability of the immunosensor was investigated by keeping it in PBS at 4 °C and after 28 days, the DPV response decreased by 10.9%. The immunosensor was applied to spiked real samples. The recoveries for chicken liver samples (97.4–103.2%) and for milk samples (96.0–104.7%) were satisfactory.

Tetracycline (TC) is a broad-spectrum antibiotic that gives its name to a whole class of antibiotics known as tetracyclines. It is commonly used for bacterial infections, respiratory and inflammatory diseases [144]. However, because of its heavy use, TC can be found in different food products such as eggs, meat, and milk products, and antibiotic resistance or allergic reactions may be triggered, as expected.

An electrochemical sensor for determining TC was assembled using a GCE modified with MnO_2_ nanoparticles (MnO_2_NPs) incorporated in a Zr-MOF [144]. The Zr-MOF served as a dispersing medium for the nanoparticles as previously indicated [87,118,143], and, in synergy with them, enhanced the conductivity, active specific area, and electron transfer from and to the sensing surface. After the optimization of the experimental conditions and using DPV, the sensor evidenced a linear detection range of 2–200 μM and a LOD of 2.577 × 10^−8^ M. The reproducibility was studied, obtaining an RSD% value of 1.52%. Considering the operational stability, after six consecutive measurements, the electrochemical signal retained 85.10% of its initial value. Milk and egg samples were tested to prove the applicability of the sensor and the corresponding recoveries ranged from 103.10 to 108.56%.

Oxytetracycline (OTC) is another broad-spectrum antibiotic belonging to the tetracycline class [145] and is used to treat different bacterial infections, but the presence of its residues in food products as meat or egg, can induce resistance to antibiotic treatment.

**Figure 12:**
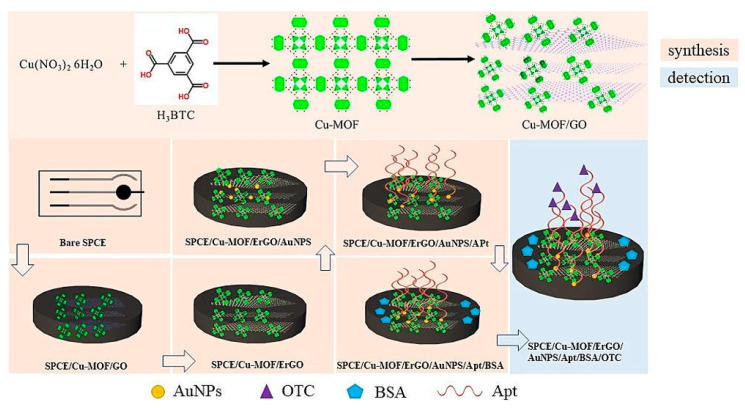
Scheme showing the composite synthesis, the sensor development and the detection approach of OTC. Reprinted with permission from [145]. Copyright 2025, Elsevier.

An aptasensor for the determination of OTC was developed based on a SPCE modified with Cu-MOF, electrochemically reduced graphene oxide (ErGO), and AuNPs [145], as shown in Figure 12.

As reported previously [87,118,143,144], Cu-MOF, as a metal-based MOF, had a large surface area, acted as a dispersing agent for AuNPs, and integrated ErGO. Moreover, the presence of AuNPs and ErGO enhanced the conductivity, so the sensor response was amplified. OTC was analyzed by CV, with a linear range (0.1–105 ng/mL) and a low limit of detection of 0.03 ng/mL. The reproducibility of the aptasensor was investigated, and an acceptable RSD of 2.47% was found. Ampicillin, doxycycline, erythromycin, chlortetracycline, kanamycin, streptomycin and chloramphenicol were tested as possible interfering antibiotics, but all of them did not affect the response of the analyte. Stability was also considered, and after 35 days at 4 °C, the electrochemical response retained 72.4% of its initial value. The aptasensor was applied to samples of milk and pork meat, with a recovery range of 87.0–110.2%. Finally, these results were comparable to those obtained by LC–MS.

Erythromycin (ERY) is a synthetic and broad-spectrum antibiotic, belonging the macrolide family [146]. However, its uncontrolled and excessive use has induced environmental pollution, consequent bacterial resistance and detrimental effects on human health, wildlife, and ecosystems.

A portable electrochemical immunosensor for the detection of ERY was prepared by modifying a SPCE using a semiconductive copper/ferric bimetallic metal–organic framework (scMOF). scMOF was synthesized starting from 2,3,6,7,10,11-hexahydroxytriphenylene (HHTP) as linking ligand (indicated as CuxFe_3_-x(HHTP)_2_) and it was a highly porous material and served as the platform for immobilizing the antibody. ERY was quantified by chronoamperometry in a concentration range from 1.0 fg/mL to 1.0 ng/mL (1.36 fM-1.36 nM), with a LOD of 0.69 fg/mL (0.96 fM). Different antibiotics such as gentamicin, kanamycin, lincomycin, tylosin and OTC were used to verify the selectivity of the immunosensor, but their responses were minimal respect of that of ERY. The reproducibility was satisfactory with an RSD value of 3.30%. The storage stability was studied and after 15 days under not specified experimental conditions, the amperometric response mostly remained unaltered. To examine the applicability of the immunosensor, spiked samples of drinking water, pork and chicken were analyzed. The following recovery range were obtained: 91.0–124.0% for drinking water, 94.0–115.9% for pork meat, and 102.6–124.0% for chicken.

Pefloxacin (PEF) is a broad-spectrum antibiotic effective against Gram-negative and Gram-positive bacteria by inhibiting DNA replication [147]. It is used for various infectious diseases in humans and animals. Its residues released into the environment can contribute to environmental pollution and the emergence of antibiotic-resistant bacteria, accumulating in the bodies of humans and animals through food products. An electrochemical sensor for detecting PEF was developed using a glassy carbon electrode (GCE) and a molecularly imprinted polymer (MIP) combined with a nanocomposite of gold nanoparticles (AuNPs) and black phosphorus nanosheets (BPNS/AuNPs) [148]. PEF served as the template molecule, while pyrrole acted as the functional monomer. AuNPs were synthesized onto BPNs, and the resulting composite was drop-cast onto the GCE, followed by electrosynthesis of PPY as MIP onto BPNS/AuNPs/GCE. PEF was analyzed via linear sweep voltammetry (LSV), resulting in a linearity range of 0.005–10 μM, with a limit of detection (LOD) of 0.80 nm. Amoxicillin, tetracycline, dopamine, ascorbic acid, uric acid, and lysine were tested as potential interferences, and the experimental results indicated that MIP could not bind all the interfering molecules. The repeatability and reproducibility were acceptable, with corresponding relative standard deviation (RSD) values of 2.89% and 1.69%, respectively. The long-term stability was assessed, and after 5 weeks of storing the sensor in a dry state and protected from light, the LSV response retained 91.7% of its initial value. Finally, PEF was analyzed in samples of milk and orange juice, with recoveries ranging from 99.16% to 102.6% for milk samples and from 95.33% to 101.5% for orange juice.

Sulfadiazine (4-amino-*N*pyrimidine-2-yl-benzenesulfonamide, SDZ) is a broad-spectrum sulfonamide antibiotic, and it is commonly utilized in both human and veterinary medicine. In the literature, it is reported that SDZ can produce liver damage, genetic damage, and resistance to antibiotics and is assumed as a contaminant with a dangerous impact on the environment and the human and animal health [148]. A nanocomposite containing zinc copper sulfide nanoparticles (ZnCuSNPs) incorporated in hydroquinone-functionalized boron nitride (f-BN) nanosheets (f-BN@ZnCuS) was used to modify a GCE and assemble an electrochemical sensor to detect SDZ [149]. The synergistic combination of f-BN and ZnCuS nanoparticles improved the properties of the sensing interface. In fact, f-BN promoted the conductivity, stability, and accelerated the electron transfer rate, whereas ZnCuS NPs provided suitable active sites for sulfadiazine adsorption. SDZ was determined by DPV, and a linearity range of 10–130 μM with a LOD of 0.008 μM was found. Common drugs, antibiotics and biomolecules, such as sulfamethazine, ibuprofen, amoxicillin, UA, ascorbic acid, DA, fumaric acid, glucose, and lactic acid, were identified as possible interferences, and none of them affected the DPV signal. The sensor evidenced an RSD of 3.96% for five successive determinations, considering the repeatability. Reproducibility was also evaluated using five independent sensors, and the corresponding RSD was 2.60%. After a storage of 30 days at 7 °C, the DPV signal decreased by 18%. Spiked milk and water samples were analyzed to investigate the applicability of the sensor to food products. The corresponding recovery range was 93.96–97.3%.

We would like to comment briefly on the examples reported above. If we go to analyze the format present in the various examples, there is no prevalent. We have very similar numbers for affinity biosensors and for sensors that use a non-biological sensing interface, such as a properly functionalized nanocomposite or a MIP. Another comment regards the introduction of graphene-like 2D nanomaterials, as MOF or black phosphorus and boron nitride nanosheets, in nanocomposites to improve the qualities of the sensing interface, and it should be pointed out how they integrate synergistically their properties with the other “ingredients” of the nanocomposite. Unfortunately, in all but one of the examples mentioned, there is no comparison with conventional methods of analysis for the analysis of foodstuffs. Such a comparison would have been important to better assess the performance of electrochemical sensors and biosensors on real samples.

Table 4 summarizes the analytical performances of the electrochemical (bio)sensors for antibiotics determination, together with the corresponding sensor formats.

## 4. Advances in Nanozyme-Based Electrochemical Sensors for Food Safety

Nanozymes are nanomaterials that exhibit enzyme-like catalytic activity, representing a rapidly advancing frontier in biocatalysis. Emerging from the convergence of nanotechnology, biochemistry, and materials science, nanozymes leverage their unique dimensions, architectures, and physicochemical properties to mimic various natural enzymes effectively. These include peroxidases (POD), catalases (CAT), oxidases (OXD), and superoxide dismutases (SOD). Compared to natural enzymes, nanozymes offer improved operational stability, reduced production costs, and tunable catalytic properties through rational structural engineering. This versatility has fueled a rapid expansion of nanozyme research, with their catalytic capabilities progressing from simple redox reactions to a broad spectrum of catalytic categories, such as hydrolytic enzymes, isomerases, and lyases. The structural diversity of nanozymes encompasses various classes of materials, including metal nanoparticles (NPs), carbon-based materials, and metal–organic frameworks (MOFs). For instance, carbon dot nanozymes have been shown to exhibit superoxide dismutase (SOD) activity, mimicking natural enzymes under physiological conditions [150]. Similarly, a range of metal nanoparticles has demonstrated biocatalytic activities suitable for electrochemical sensor design [151]. The catalytic mechanisms of nanozymes typically involve electron transfer reactions akin to those performed by natural enzymes. This unique characteristic enables nanozymes to facilitate biochemical reactions across a spectrum of conditions, making them highly promising for food safety applications [152]. The catalytic mechanism of nanozymes is generally controlled by surface-mediated reactions that involve the generation of reactive oxygen species and electron transfer processes, closely paralleling the active sites found in natural enzymes [153]. A given nanozyme can exhibit multiple enzyme-mimicking activities, with those activities being tunable by modifying particle size, morphology, and surface chemistry. Variations in composition, size, and morphology significantly influence their catalytic efficiency [154]. Moreover, the presence of specific active sites, comparable to those in natural enzymes, is key to nanozyme action. Complex interactions between substrate molecules and nanozymes allow them to catalyze reactions effectively, providing performance comparable to traditional enzymes [155]. Modulations in the coordination environment and the creation of defect sites on the nanozyme surface also pave the way for enhancing reactivity and substrate recognition, which is essential for the detection of specific foodborne contaminants. These mechanistic insights are crucial for designing next-generation sensors with lower limits of detection and improved analytical reliability.

The integration of nanozymes into electrochemical biosensors represents a central and rapidly developing area of contemporary sensor research. This convergence leverages the robust physicochemical characteristics of nanozymes, particularly their impressive electrocatalytic prowess. Their intrinsic catalytic properties, often involving electron transfer during substrate oxidation, are critical for their efficient operation in electrochemical systems. To enhance their performance even more, nanozymes are frequently engineered as nanocomposites, often through doping specific elements or incorporating carbon nanostructures. These modifications significantly improve electrical conductivity and catalytic efficiency. Such design improvements not only broaden substrate specificity but also extend their stability under harsh conditions, which is especially crucial for complex analytical applications in diverse matrices [152].

The introduction of nanozymes is changing biosensing, with a particular emphasiz on food safety. In this field, the accurate and rapid detection of contaminants such as toxins, foodborne pathogens, pesticides, and antibiotics is paramount. Nanozymes offer significant advantages over natural enzymes in food analysis due to their enhanced stability and tunable activity, making them highly attractive for this domain. Advanced design strategies have enabled nanozymes to participate in cascade reactions and synergistic amplification systems. These innovations significantly enhance the sensitivity and selectivity of electrochemical biosensors for food contaminants. Consequently, nanozymes are actively redefining analytical methodologies in food analysis by either replacing or complementing natural enzymes, thereby addressing the critical need for rapid and highly sensitive detection methods.

Recent literature highlights the growing significance of nanozyme chemistry in food safety, emphasizing advancements in synthesis, surface modification, and integration into electrochemical biosensing platforms. These developments harness the catalytic properties of nanozymes to enable diverse, sensitive, and selective strategies for contaminant detection.

### 4.1. Mycotoxins

Three recent interesting studies addressed the detection of mycotoxins such as deoxynivalenol (DON) [156] and its masked form deoxynivalenol-3-glucoside (D3G) [157], and aflatoxin B1 (AFB1) [158], illustrating the common use of nanozymes as signal amplification elements in electrochemical sensing platforms. While each study employed distinct strategies for target recognition (immunosensors, aptasensors, MIPs), signal generation, and system design, all leveraged nanozyme-catalyzed redox reactions to amplify signals and generate measurable electrical currents. A shared feature among the nanozymes in all three systems was their pronounced peroxidase-like activity, which was essential for signal generation, either by catalyzing the H_2_O_2_-driven oxidation of thionine [157] or by facilitating electron transfer processes [157,158]. Table 5 summarizes the key features of the nanozyme-based electrochemical sensors developed.

Each study displayed the applicability of their sensor in real food samples. These diverse approaches demonstrated the versatility of nanozymes in addressing complex challenges in food contaminant analysis, paving the way for advanced and practical biosensing technologies.

### 4.2. Antibiotics

Two recent studies demonstrated the use of nanozyme-based electrochemical aptasensors for detecting antibiotic residues: tetracycline [159] and aminoglycoside antibiotics (AAs) [160].

**Figure 13:**
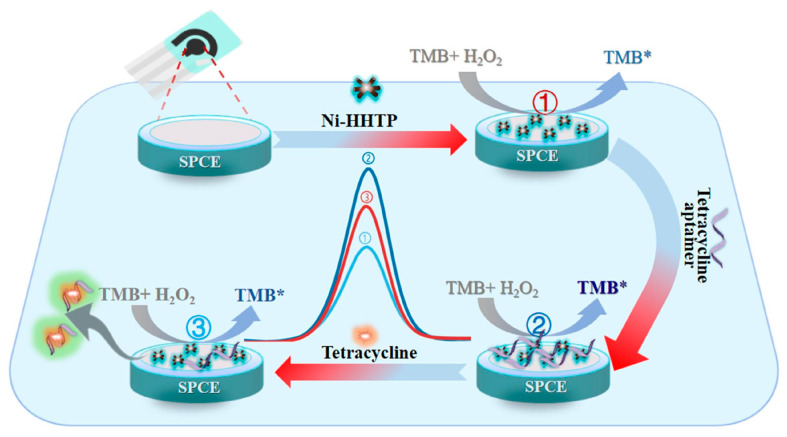
Scheme showing the sensor development and the detection approach of TC. Reprinted with permission from [159]. Copyright 2024, Elsevier.

In the first study, researchers employed a nickel-based metal–organic framework nanozyme (Ni-HHTP), which demonstrated intrinsic peroxidase-like activity [159], as illustrated in Figure 13.

When deposited on a screen-printed electrode, Ni-HHTP was functionalized with a TC-specific aptamer (TC-Apt), acting both as a recognition element and as an enhancer of the nanozyme’s catalytic activity. Detection relied on variations in this activity upon TC binding, using TMB and H_2_O_2_ as substrates. The resulting electrochemical response facilitated quantitative analysis across a concentration range of 10 pM to 1.0 μM, with a detection limit of 1.9 pM.

In the second study, a dual-mode aptasensor for the sensitive detection of aminoglycoside antibiotics (AAs) was described, featuring a colorimetric and an electrochemical mode that employed a broad-spectrum aptamer and Au-Pd@Fc nanoprobes functionalized with signal DNA [160]. Detection relied on an exonuclease III-assisted cyclic amplification strategy, where target-induced cleavage promoted probe accumulation on the electrode, amplifying the signal. Designed for AA quantification in milk, the sensor achieved a low detection limit of 0.0355 ± 0.00613 nM and accurately identified 10 AAs, with average recovery rates of 97.19 ± 4.41%. Although the limitations of the electrochemical mode were not discussed, the study attributed the high sensitivity to the effective signal amplification mechanism, underscoring its potential for rapid and accurate food safety monitoring.

Both papers emphasized that their sensors were designed to overcome challenges posed by complex food matrices and highlighted the crucial role of rational design in developing highly sensitive and specific aptasensors for detecting antibiotic residues in food.

### 4.3. Foodborne Pathogens

Recent research [161,162] highlighted significant advancements in developing highly sensitive and selective electrochemical biosensors for bacterial pathogen detection. Their approach leveraged the synergistic combination of bacteria-imprinted polymers for selective pathogen recognition and manganese dioxide (MnO_2_) nanozymes for signal amplification through catalytic activity, demonstrating versatile applications in enhancing electrochemical readouts for microbial identification.

The first study focused on single-cell level detection of *Staphylococcus aureus* in spiked milk samples using a sensor incorporating bacteria-imprinted polymers (BIPs) and vancomycin-conjugated MnO_2_ nanozymes. The sensor’s exceptional sensitivity and specificity were ascribed to the synergistic effects of the molecular imprinting technique, which improved bacterial recognition, and the catalytic properties of the MnO_2_ nanozyme, which facilitated direct electron transfer, producing strong electrochemical signals. This sensor was validated in complex biological samples and successfully detected S. aureus at concentrations as low as 10 CFU/mL, exhibiting excellent selectivity, particularly identifying target *S. aureus* from interfering bacteria of the same genus at concentrations 100-fold higher. These findings indicated its potential for applications in meat and dairy product safety, where *S. aureus* contamination poses a significant risk [161].

**Figure 14:**
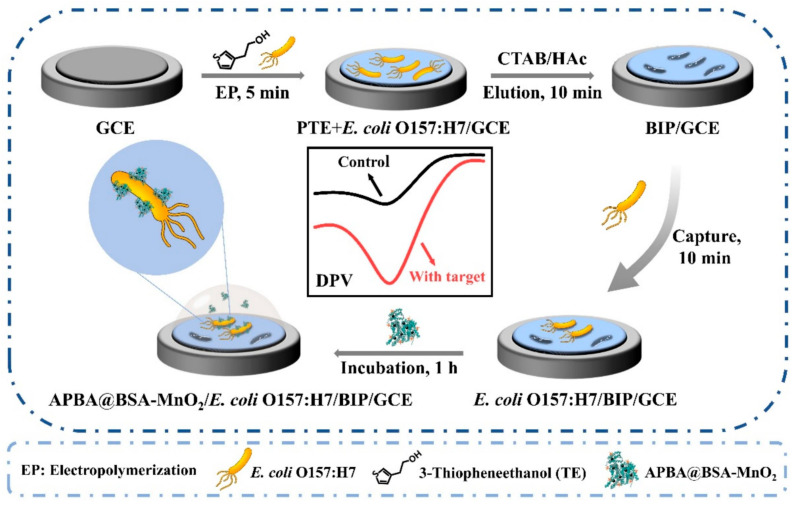
Scheme illustrating the sensor development and the detection approach of *E. coli.* Reprinted with permission from [162]. Copyright 2025, Elsevier.

Starting from these significant results, a sandwich-type electrochemical sensor for the sensitive and selective detection of *Escherichia coli* O157:H7 in spiked milk samples was developed [162], as shown in Figure 14.

This sensor integrated a BIP for specific bacterial capture with a signal amplification strategy utilizing 3-aminophenylboronic acid-conjugated BSA-templated MnO_2_ nanozymes (APBA@BSA-MnO_2_). The BIP provided selective recognition and capture of *E. coli* O157:H7, as previously reported for *Listeria monocytogenes* [92], while the MnO_2_ nanozyme catalyzed the reduction of hydrogen peroxide (H_2_O_2_), generating a measurable electrochemical signal. The sensor achieved a detection limit of 10 CFU/mL and effectively detected *E. coli* O157:H7 in complex milk matrices at the same concentration, requiring only a simple 10-fold dilution for sample pretreatment. Moreover, it displayed exceptional selectivity, successfully identifying *E. coli* O157:H7 in the presence of the closely related serotype *E. coli* O6, even at 100-fold higher concentrations [162]. Both studies successfully highlighted the power of integrating nanozyme catalysis with molecular recognition, demonstrating a robust, cost-effective, and practical approach to real-world microbial detection without explicitly discussing limitations. These strategies collectively illustrate the evolving landscape of nanozyme-enabled biosensors for critical applications in food quality control and safety assurance.

### 4.4. Pesticides

The development of nanozyme-based electrochemical sensing platforms for the detection of pesticide residues, primarily in fruit and vegetable samples, has been also described in recent literature. Specifically, one study targeted the dithiocarbamate fungicide thiram [163], three of the studies [164,165,166] focused on organophosphate pesticides, and one also on carbamate pesticides carbofuran and carbosulfan [166]. The effectiveness of the developed sensors was validated through assessments of analytical performances and applicability in real-world samples (Table 6).

The various research groups adopted distinct yet innovative sensing mechanisms to leverage nanozyme capabilities for pesticide detection. A ratiometric dual-signal approach was described by [164], where the binding of thiram (THR) to their nanozymes simultaneously inhibited catechol oxidation and enhanced the nanozymes’ inherent electrical conductivity. This unique interplay allowed for a self-calibrating signal based on the ratio of two distinct currents. In contrast, refs. [164,165,166] all employed an enzyme inhibition mechanism, primarily utilizing acetylcholinesterase (AChE). In these systems, the presence of organophosphate pesticides inhibited AChE activity, which, in turn, modulated the catalytic activity of their respective nanozymes and the resulting electrochemical signal. More specifically, refs. [164,165] linked AChE inhibition to the production of thiocholine (TCh). TCh then affected the peroxidase-like activity of their nanozymes (NiCoFeS/rGO and cobalt-doped Ti_3_C_2_ MXene nanosheets, respectively) in the oxidation of *o*-phenylenediamine (OPD). While ref. [166]’s system also relied on cascaded catalytic reactions involving AChE inhibition, the precise details of how the single-atom Ce-N-C nanozyme (SACe-N-C) signal was modulated in their study were not explicitly detailed in the same manner as the TCh-mediated mechanisms. These mechanistic differences also highlighted the unique contributions of each study to the field. In particular, one work [164] addressed a notable gap by emphasizing the under-explored potential of nanozymes’ electrochemical properties beyond their traditional role in catalyzing the production of electroactive substances. Another work highlighted their SACe-N-C nanozyme as a significant advancement in overcoming the common limitation of low catalytic activity in conventional nanozymes [166]. Meanwhile, another innovation involves the in situ generation of electroactive substances within their homogeneous electrochemical (HEC) sensor, a breakthrough that effectively eliminated high background signals and false positives often associated with traditional HEC sensors [165].

## 5. Conclusions, Challenges, and Future Perspectives

The development of sensitive, reliable, robust, portable, and cost-effective sensing approaches has become essential for ensuring food safety. This addresses the critical issue of contamination and infection of food products caused due to bacteria, contaminants, pesticides and antibiotics.

Considering the disadvantages of conventional analytical methods, such as complex procedures, high costs, lengthy analysis times, and elaborate sample pretreatment, it is evident that electrochemical (bio)sensors offer a very attractive alternative because they are easy to handle, relatively affordable, sensitive, and easily miniaturized. However, several issues and challenges must be addressed. For example, in some cases complex procedures are introduced for the sensor assembling, including the synthesis of the sensing electrodic materials or the use of sensitive reagents. They can lengthen the actual measurement times, but can also be expensive, making them unsuitable for their application for food safety and their commercialization

In this section, some comments regarding the role of the different materials, electrodes and (bio)sensors typologies, together with some remarks on the analytical performances, are summarized. Finally, challenges and future perspectives on the electrochemical sensing approach for food safety are introduced and evidenced.

First, analyzing the format of sensors for different types of contaminants reveals that there is no prevailing format for all contaminants. In fact, we can underline that the choice of format depends on the target analyte. In the case of toxins, the predominant format is aptasensor (see Table 1), but for foodborne bacteria, we have an equal number of aptasensors, immunosensors and genosensors (see Table 2). Turning to pesticides, we have a good number of enzymatic biosensors along with a comparable number of electrochemical sensors with a recognition element, such as MIP or a nanocomposite (see Table 3). Finally, for the antibiotics, we have very similar numbers for affinity biosensors and for sensors that use a non-biological sensing interface, such as a properly functionalized nanocomposite or a MIP (see Table 4).

On the other hand, examples of electrochemical sensors where the recognition element is represented by a molecularly imprinted polymer are rather limited, although the analytical performance of the corresponding sensors is very promising. We would like to highlight several examples of biosensors based on phages. They can serve as a valid alternative to genosensors and immunosensors because they are accurate and highly specific towards the target analytes compared to other biorecognition elements such as aptamers or antibodies. Moreover, phages are certainly less expensive, can be easily functionalized, and are quite stable regardless of the experimental conditions.

Nanomaterials, nanoparticles or nanocomposites are used to improve the characteristics of sensing interfaces in terms of conductivity and electron transfer rate and to amplify the electrochemical signal. We must highlight the widespread use in the examples of 2D nanomaterials and not just graphene or graphene oxide. We are mainly talking about TMDs, COFs, MOFs and MXenes together with g-C_3_N_4_. TMDs and MXenes exhibit good conductivity, thermal stability and are easily functionalized, while COFs and g-C_3_N_4_ are stable and easily functionalized, but evidence lower conductivity; therefore, generally metal nanoparticles such as AuNPs can be incorporated into their structure. AuNPs are the most used nanoparticles because of their well-established synthetic protocol. In general, we can conclude that the integration of nanomaterials with other nanomaterials, with either natural polymers such as chitosan or synthetic is the best option to have an optimal platform sensing with adequate conductivity and stability, equipped with functional groups for the immobilization of biomolecules, where appropriate and with an ad hoc designed nanoarchitecture.

Considering the electrode typologies reported in this review (see Table 1, Table 2, Table 3 and Table 4), different types of electrodes are employed, from the more conventional, such as GCE, AuE, and ITOE, to SPEs, among others. GCE was the preferred option, probably because of its well-known chemical–physical properties [1]. AuE and SPEs were employed in several examples because of the biocompatibility, stability, easy functionalization, and conductivity of Au and SPEs represent a low-cost solution for the sensing platforms, above all for the development of portable devices.

We should now examine the analytical performance of sensors. The corresponding linearity ranges, and the LODs seem to be very promising, being generally below the required levels in current contaminant regulations in Europe, America and Asia.

The selectivity of the sensor is generally investigated, but the criteria for the selection of interfering molecules are not obvious. The selection criteria may be different, such as similarity in structure, analogy of use, belonging to the same class or family of chemical compounds, or finally, presence in the same complex matrix to be analyzed. The choice criterion must be indicated otherwise, it will not be possible to compare sensor performance with the same or structurally similar targets.

The reproducibility and the stability of the sensors are usually addressed, but it is difficult to compare sensors with the same format and target when storage conditions to define sensor stability are not comparable or even unspecified.

Conventional methods of analysis have not always been used to validate sensors, and therefore, very often, a comparison is missing, which would allow us to establish whether the sensor could effectively replace the standard method.

In addition, as in the past, it must be emphasized that there are no commercially available sensors [1,8,18,29,30,37]. This is because very often the synthesis procedure of the materials involved is complex and in some cases, expensive. In addition, there may be problems in preparing real samples, or due to the presence of interfering molecules or unexpected side reactions. Finally, the problem of fouling of the sensing surface is not adequately addressed.

In summary, several challenges relate to the stability of the biorecognition element, the cost of materials used, and the suitability of testing in complex real matrices. The realization of on-site analysis and accurate assessment of the actual matrix can partly address these issues, along with the miniaturization of biosensing devices, as well as the proper integration of nanomaterials or nanocomposites.

Chiral contaminants analysis is both a challenge and a promising perspective for the future [167]. The determination of enantiomers in food can certify the products authenticity and guarantee their safety. Amino acids and chiral pesticides are important chiral compounds in food. Pesticides are generally used in a racemic formulation, but it would be useful to have detailed and accurate information on the toxicity on an enantiomeric basis, not only on a racemic basis. In this way, it would be possible to identify whether one of the two enantiomers may be more toxic to human and animal health. In analogy with the literature for amino acids [167,168], the realization of a chiral electrochemical sensor must include an appropriate chiral selector deposited on the electrode surface that can interact and detect the enantiomer to be determined.

Finally, we would like to add some comments regarding the nanozyme-based electrochemical sensors. Despite their promising potential in ensuring food safety, the widespread adoption of nanozyme-based electrochemical sensors remains limited by several critical challenges that must be addressed to enable their broader implementation. A primary concern is production costs and scalability. The reliance on sophisticated nanomaterials and complex synthesis methods often leads to high manufacturing expenses, hindering broader accessibility.

Streamlining these processes and sourcing more economical raw materials are crucial steps toward cost-effective, large-scale production. In this context, microbial nanobiosynthesis presents a particularly promising approach, a green and sustainable method for producing nanomaterials using microorganisms such as bacteria and microalgae [169,170]. Fungi also show great potential for this purpose [171]. Another technical challenge includes the control of the signal amplification. While this **is** essential for the sensors’ sensitivity, uncontrolled amplification can result in unreliable or inaccurate readings, particularly in complex food matrices.

Precise tuning of amplification mechanisms **is** imperative to ensure consistent and reproducible sensor outputs across diverse food samples, preventing issues like signal instability or overshooting [172]. Furthermore, safety and environmental concerns surrounding the nanomaterials used in these sensors cannot be overlooked. The potential for nanoparticle toxicity and accumulation in both humans and ecosystems necessitates the development of biocompatible and environmentally friendly nanozymes. Ensuring that these materials pose no risks throughout their lifecycle, from production to disposal, is vital for sustainable sensor development and consumer confidence in food safety applications [173].

The field is poised for significant advancements, driven by breakthroughs in material science, interdisciplinary collaboration, and digital innovation, which will greatly benefit food safety applications. Future efforts will focus on refining nanozyme design to enhance catalytic efficiency, structural stability, and target specificity, making them more resistant to diverse food matrices. Integrating machine learning and computational modelling promises to enable the rational design of nanozymes with tailor-made functionalities for specific food contaminants [174].

## Figures and Tables

**Table 1:** Analytical performances and format of electrochemical (bio)sensors for toxin determination.

Electrode	Format	Technique	Sample	Linearity	LOD	Recovery %	Reference Method	Ref.
PGE	Electrochemical sensor	DPV	SEN/flour, herbal tea	25–125 μg/mL	5.45 μg/mL,	-	-	[46]
GCE	Electrochemical sensor utilizing MIP/Fe_3_O_4_/GO/GCE	SWV	PAT/apple and pear juice	0.001 nM–250.0 nM	3.33 × 10^−4^ nM	94.0–103	-	[53]
Pt	Microfluidic immunosensor based on	A	T-2/wheat samples	0–1000 μg/kg	0.10 μg/kg	97.4–101.6	ELISA	[54]
SPCE	Bio-3D-printed liver microtissue biosensor based on COX/AuNPs/AAO/SPCE	CV	DON/-	2~40 μg/mL	1.229 μg/mL	-	-	[57]
GCE	Aptasensor based on MCH/Apt/AuNPs/ZIF-8/GCE	DPV	AFB1/corn and peanut oil	10.0–1.0 × 10^5^ pg/mL	1.82 pg/mL	93.49–106.9	-	[60]
AuE	Bifunctional genosensor based on TDNs/HPG/AuE	DPV	AFB1/peanut OTA/peanut	0.05–360 ng/mL 0.05–420 ng/mL	3.5 pg/mL 2.4 pg/mL	96–102 99–102		[61]
SPCE	Aptasensor based on Au NPs@Ce-TpBpy COF	CA	ZEN/corn flour	1 pg/mL–10.0 ng/mL	0.389 pg/mL	93.0–104.7		[63]
GCE	Electrochemical sensor based on Bi_2_S_3_@CNF/GCE	A	ZEN/wheat and oats	0.125–375.5 μM 438–1951 μM	0.61 μM	98.9–99.15	-	[65]
GCE	Dual-signal ratiometric electrochemical aptasensor based on MoS_2_-Thj	DPV	ZEN/corn flour	1.0 × 10^−10^–1.0 × 10^−6^ M	4.4 × 10^−11^ M	99.4–109.5	-	[66]
GCE	Aptasensor based on HP1-MB/AuNPs/GCE	SVW	ZEN/corn flour, peanut oil, and wine	100 fg/mL–50 ng/mL	89 fg/mL	93.52–110.85	HPLC	[67]
AuE	Aptasensor based on E-AB/AuE	SWV	MC-LR/tap and pond water	1.0–750.0 ng/L	0.53 ng/L	96.11–105.60	-	[69]
SPCE	Immunosensor based on anti-MC-LR/MC-LR/cysteamine/SPCE	EIS	MC-LR/surface water	0.1–100 μg/L	0.69 ng/L	-	ELISA	[70]
SPCE	Electrochemical biosensor based on Specific Binding Peptide/PPy/AuNPs/SPE	EIS	TTX/-	2–1000 ppb	2.80 ppb	-	-	[71]
SPAuE	Electrochemical sensor based on MIP/SPAuE	DPV	TTX/mussel	5.0–25.0 μg/mL	1.14 μg/mL	81.0–110.2	HPLC-TMS	[72]
SPCE	Aptasensor based on CHI/AuNPs	CV	OA/mussel and scallop	0.01–100 ng/mL	6.7 pg/mL	92.3–116	-	[74]

**Table 2:** Analytical performances and format of electrochemical (bio)sensors for foodborne pathogens determination.

Electrode	Format	Technique	Sample	Linearity	LOD	Recovery %	Reference Method	Ref.
Nylon threads	Microfluidic aptasensor based on-PLL MoS_2_/Nylon threads	DPV	VP/seafood	10–10^6^ CFU/mL	5.74 CFU/mL	-	Counting plate	[76]
GCE	Receptor-binding phage proteins RBPP-based biosensor, including MWCNTS and PBSE	EIS	CJ/chicken cecal	10^2^–10^9^ CFU/mL	10^2^CFU/mL		-	[77]
SPCE	Electrochemical sensor based on MIP	EIS	PSA via BHL/Tap water	10–1 × 10^3^ nM	31.78 nM	.	.	[80]
Si mold coated with Cr and Au	Immunosensor based on 3D multilevel micro/nano protrusions, including Au nanoclusters	EIS	ST/milk	10–10^5^ CFU/mL	10 CFU mL	-	Counting plate	[83]
AuIDE	Genosensor using CRISPR/Cas9 system and rolling circle amplification (RCA)-assisted “silver chain”	EIS	ST/milk, beef, fish, lettuce, bean skin	10–10^7^ CFU/mL	7 CFU/mL	90.63–113.00	-	[84]
SPCE	Genosensor based on RPA-CRISPR/Cas12a	DPV	ST/-	1.04–1.04 × 10^8^ CFU/mL	3 CFU/mL			[86]
SPCE	Aptasensor based on GQDs/Cu-MOF nanocomposite	DPV	ST/tap and river water, milk, *Lonicera japonica*	5.0–5.0 × 10^8^ CFU/mL	0.97 CFU/mL	97.30–106.80	ELISA	[87]
SPCE	Portable immunosensor based on 3D-NPS-doped CNSs	DPV	ST/tap water, guava juice	1.0 × 10^2^–5.0 × 10^2^ CFU/mL	24 CFU/mL	-	ELISA	[88]
GCE	Aptasensor based on Si@MB and AuNPs	DPV	L.M./lettuce, fresh-cut fruit	10^2^–10^7^ CFU/mL	2.6 CFU/mL	80.0–116.0	Counting plate	[90]
GCE	Genosensor based on SRCA and NEMA, including AuNPs	DPV	L.M./food samples	5.4–5.4 × 10^7^ fg/μL	2.13 fg/μL	91.4–111.1	RT-qPCR	[91]
GCE	Aptasensor based on MIP and BUHNPs	DPV	L.M./drinking water, orange juice	10–10^6^ CFU/mL	1.0 CFU/mL	90.2–105.9	-	[92]
GCE	Immunosensor based on PDA@ZnMoO4/MXene nanocomposite	DPV	L.M./milk, smoked seafood	10–10^7^ CFU/mL	12 CFU/mL	98.0–126.0		[93]
ITOE	Electrochemical sensor based on MIP	A	SA/water	1–10^8^ CFU/mL	3.42 CFU/mL	96.94–108.25	-	[95]
AuE	Genosensor based on PCR and CRISPR/Cas12a	DPV	SA/poultry	67–6.7 × 10^5^ CFU/mL	55 CFU/mL	-	-	[96]
GCE	Genosensor based on MoS_2_@CNT/CHIT nanocomposite	DPV	SA/water, milk	1.0 × 10^4^–1.0 × 10^11^ CFU/mL	1.0 × 10^4^ CFU/mL	92.05–99.58	-	[97]
BC	Electrochemical biosensor based on BC/PPy/RGO-phage	DPV	SA/milk, chicken	1–10^7^ CFU/mL	1 CFU/mL	97.7–99.5	Counting plate	[98]
AuE	Immunosensor based on Fe_3_O_4_@PB nanocomposite	DPV	SA/milk	73.75–7.375 × 10^7^ CFU/mL	9.912 CFU/mL	99.74–106.40	Counting plate	[99]
GCE	Aptasensor based on Apt/CS/PDA/PXA	DPV	SA/milk, orange juice	10–10^7^ CFU/mL	3CFU/mL	-	-	[100]
GCE	Immunosensor based on Co/Zn-ZIF-8–400@C-MWCNTs nanocomposite	DPV	SA/milk	1.3 × 10^2^–1.3 × 10^8^ CFU/mL	94 CFU/mL	94.07–105.76	-	[103]
ITO	Immunosensor based on VSe_2_ nanosheets	DPV	SA/sugarcane	13–10^7^ CFU/mL	0.096 CFU/mL	96.2–99.0	-	[104]
SPAuE	Electrochemical MIP sensor	CV	SA/milk, pork	10–10^5^ CFU/mL	10 CFU/mL	-	-	[105]
SPAuE	Immunosensor using a SAM	EIS	SA/-	10–10^6^ CFU/mL	10 CFU/mL	-	-	[106]
GCE	Electrochemical biosensor based on phage-encoded protein RBP 41, GO and AuNPs	DPV	SA/milk, lettuce	3–10^6^ CFU/mL	0.2984 Log_10_ CFU/mL	84.0–120.0	-	[107]
GCE	Genosensor based on CRISPR/Cas12a and CG@MXene nanocomposite	DPV	SA/chicken	1.6 × 10^2^–1.6 × 10^7^ CFU/mL	160 CFU/mL	100.46–106.37	-	[108]
AuE	Immunosensor based on Fe_3_O_4_–IL composite	DPV	SA/milk	3.65 × 10^2^–3.65 × 10^8^ CFU/mL	1.12 × 10^2^ CFU/mL	99.40–110.13	-	[109]
GCE	Electrochemical biosensor based on CFGO and CB	EIS	*E. coli*/milk, pork	10^2^–10^7^ CFU/mL	11.8 CFU/mL	60.8–114.2	-	[112]
AuE	Aptasensor using the triple helix DNA	SVW	*E. coli*/water, milk	100–1.0 × 10^7^ CFU/mL	5.2 CFU/mL	95.76–101.20	-	[114]
AuE	Electrochemical biosensor based on engineered antimicrobial peptide	DPV	*E. coli*/milk	10–10^5^ CFU/mL	3.4 CFU/mL	89.1–122.0		[115]
AuE	Genosensor involving Fe_3_O_4_@COF-AuNPs nanocomposite and TICA	CV	*E. coli*/orange juice, milk	10^2^–10^9^ CFU/mL	10 CFU/mL	92.0–109.0	-	[116]
SPCE	Aptasensor using AgNPs	CV	*E. coli*/milk, tap water	34–3.4 × 10^6^ CFU/ml	34 CFU/mL			[117]
SPCE	Aptasensor based on 2D Zn-MOFs and 2D C-Ti_3_C_2_T_x_ composite	DPV	*E. coli*, ST, SA/milk, egg	10–10^6^ CFU/mL	E. coli 6 CFU/mL STA 5 CFU/mL SA 5 CFU/mL	E. coli 82.54–132.0	Counting plate	[118]

**Table 3:** Analytical performances and format of electrochemical (bio)sensors for pesticide determination.

Electrode	Format	Technique	Sample	Linearity	LOD	Recovery %	Reference Method	Ref.
GCE	Electrochemical sensor based on Cu-PZDA/CNFs	DPV	GLP/beetroot juice, lettuce extracts	0.01–200 μM	3.12 μM	98.01–101.83	HPLC-	[123]
ITOE	Electrochemical sensor based on Co_3_O_4_ electrochromic film and MIP	CV	Deltamethrin/tomato, grapefruit, salad, orange	2.82–56.5 nM	1.53 nM	97.20–105.33	HPLC	[124]
GCE	Electrochemical sensor based on Ag NWs@MoS_2_ nanocomposite	SWV	TBZ/pear, apple	0.05–10 μM	1.75 nM	95.5–103.6%	HPLC	[125]
PCL fibers	Electrochemical sensor based on PCL/PPy/β-CD composite	DPV	DNF/rice	0.2–5 μM 5–50 μM	0.05 μM	96.67–103.65	-	[126]
GCE	Electrochemical sensor based on Co(OH)_2_/TiO_2_ composite	CV	CBZ/apple, orange, cabbage, carrot, and tomatoes	0.039–0.399 μM 0.399–2630.1 μM	0.007 μM	96.84–106.6	-	[127]
GCE	Electrochemical sensor based on CaZrO_3_@g-C_3_N_4_ composite	DPV	DFC/strawberry, grapes, spinach, and apple	0.01–230.04 μM	1.8 nM	98.20–99.80	-	[128]
GCE	Biosensor based on MXene/AuPt	DPV	CFP/apple, cabbage	10^−8^–10^−3^ mg mL	1.55 pg/mL	95.44–102.81	-	[130]
SPCE	Biosensor based on CuNWs/rGO	CV	CFP/drinking water, orange juice	10–200 μg/L	3.1 μg/L	96.67–105.65	-	[131]
GCE	Biosensor based on CHI/Pt/MoS_2_/TM	DPV	CFP/strawberry, pakchoi, Chinese chive	10^−12^–10^−6^ M	4.71 × 10^−13^M	94.81–104.0	-	[132]
GCE	Biosensor based on TiO_2_-NRs/AuNPs/CHIT@rGO	DPV	DDPV/cabbage orange juice	2.26–565 nM	2.23 nM	90.3–101.6	-	[133]
SPCE	Biosensor based on PB/CBs	CA	DDPV/apple and orange peels	0 up to 20 ppb	1nM (0.3 ppb)	Apple 106–97 Orange 91–115	-	[134]
GECE	Electrochemical sensor based on GQDs@MIP (MAL) GQDs@MIP (CBZ)	DPV	MAL, CBZ/cucumber, tomato, grape juices	MAL 0.02–55.00 μM CBZ 0.02–45.00 μM	MAL 2 nM CBZ 1 nM	97.75–109.6	-	[135]
GCE	Electrochemical sensor based on B-CuO/g-C_3_N_4_	SWASV	MAL/apple, tomato	0.18–5.66 pg/mL	1.2 pg/mL	87.64–120.59	-	[136]
GCE	Electrochemical sensor based on Ti_3_C_2_T_x_/MWCNT-OH	DPV	Paraoxon-ethyl/red and green grapes	1–100 μM	10 nM	-	-	[137]
GCE	Biosensor based on ZnO-rGO	DPV	MP/cucumber, apple	0.01–1000 ng/mL	0.00463 ng/mL	90.92–108.4		[138]
GCE	Biosensor based on MXene/MoS_2_@AuNPs	DPV	Phoxim/winter jujube, red date, raisin, and apricot	1 × 10^−13^–1 × 10^−7^ M	5.29 × 10^−15^ M	99–107	HPLC	[139]

**Table 4:** Analytical performances and format of electrochemical (bio)sensors for antibiotics.

Electrode	Format	Technique	Sample	Linearity	LOD	Recovery %	Reference Method	Ref.
AuE	Aptasensor based on Exo III and metal ion-dependent DNAzyme recycling and HCR	SWV	Tobramycin/milk	5 nM~1 μM	3.51 nM	97.6–102.7	-	[142]
AuE	Immunosensor based on Ce-MOF@AgAuNPs nanocomposite	DPV	MON/chicken liver, milk	0.05–250 ng/mL	0.008 ng/mL	97.4–103.2 in chicken liver 96.0–104.7 In milk	-	[143]
GCE	Electrochemical sensor based on MnO_2_@ Zr-MOF	DPV	TC/milk, egg	2–200 μM	2.577 × 10^−8^ M	106.26–115.01	-	[144]
SPCE	Aptasensor based on AuNPs/ErGO/Cu-MOF	CV	OTC/milk, pork meat	0.1–10^5^ ng/mL	0.03 ng/mL	87.0–110.2	LC–MS	[145]
SPCE	Immunosensor based on scMOF	CA	ERY/drinking water, pork, chicken	1.0 fg/mL–1.0 ng/mL	0.69 fg/mL	91.0–124.0 in drinking water, 94.0–115.9 in pork meat 102.6–124.0 in chicken	-	[146]
GCE	Electrochemical sensor based on MIP/BPNS/AuNPs	LSV	PEF/milk, orange juice	0.005–10 μM	0.80 nM	99.16–102.6 in milk 95.33–101.5 in orange juice	-	[147]
GCE	Electrochemical sensor including f-BN@ZnCuS nanocomposite	DPV	SDZ/milk water	10–130 μM	0.008 μM	93.96–97.3	-	[149]

**Table 5:** Summary of three recent studies on nanozyme-based electrochemical sensors for detecting DON, D3G, and AFB1 in food safety applications.

Target Analyte and Samples	Recognition Strategy	Nanozyme Properties and Multifunctionality	Signal Generation and Measurement	LOD	Reported Limitations/Future Directions	Ref.
DON in spiked corn, wheat, flour and rice	Label-free electrochemical immunosensor (antibody–antigen interaction)	Ni-Fe PBA nanozymes with strong peroxidase-like activity	H_2_O_2_-driven oxidation of thionine catalyzed by nanozymes; signal inversely related to DON concentration	0.005 ng/mL	No explicit limitations mentioned	[156]
D3G in wheat and barley	MIP-based electrochemical sensor using molecular imprinting for selective recognition	Mn-CeO_2_ nanozymes with implied peroxidase-like activity, used primarily for signal amplification	Electrochemical signal amplified by Mn-CeO_2_ nanozymes; mechanism not explicitly detailed but likely involves catalytic oxidation reactions	0.003 ng/mL	Identifies need for evaluation of interference, stability, reusability, and reduction of sample prep complexity	[157]
AFB1 in peanut	Dual-signal EBFC system with aptamer-functionalized nanozymes	CoMn-CeO_2_ nanospheres with both glucose oxidase- and peroxidase-like activities	Signal 1: Reduced glucose oxidation due to aptamer-nanozyme release. Signal 2: Precipitate formation impeding electron transfer (via peroxidase activity); self-powered system	5.8 pg/mL	No explicit drawbacks mentioned	[158]

**Table 6:** Summary of recent studies on nanozyme-based electrochemical sensors for detecting pesticides in food matrices.

Target Analytes and Applications	Nanozyme Composition and Design	Sensing Mechanism and Signal Transduction	LOD	References
Thiram in fruit and vegetable, samples (Pear, apple, broccoli, and cucumber)	MOF-derived electroactive nanozymes	Ratiometric electrochemical sensor with dual-signal response: THR suppresses catechol oxidation and enhances nanozyme conductivity, enabling self-calibrated detection	0.15 nM	[163]
Trichlorfon in fruits and vegetables (plums, watermelon, lettuce and cucumber)	NiCoFeS/reduced graphene oxide (rGO) nanozyme	AChE inhibition modulates nanozyme peroxidase-like activity, altering electroactive product (e.g., OPDox) generation	9.74 fg/mL	[164]
Paraoxon in Pak-Choi (Chinese cabbage)	Cobalt-doped Ti_3_C_2_ MXene nanosheets (CMNSs)	AChE inhibition affects peroxidase-like activity; in situ electroactive signal generation without external reagents	0.02 ng/mL	[165]
Omethoate, methamidophos carbofuran, and carbosulfan in fruits and vegetables (broccoli, ginger, rape, celery, baby bok choy, Chinese cabbage, apple, and tomato)	Single-atom Ce-N-C nanozyme (SACe-N-C)	AChE inhibition affecting peroxidase-like activity of the nanozyme, changing electrochemical signals	Omethoate (55.83 ng/mL), methamidophos (71.51 ng/mL), carbofuran (81.81 ng/mL) carbosulfan (74.98 ng/mL)	[166]

## Data Availability

Not applicable.

## Abbreviations

The following abbreviations are used in this manuscript:

3D-NPS-doped CNS	3D nitrogen-, phosphorus-, and sulfur-doped carbon nanosheets
A	Amperometry
AA	Ascorbic acid
AAs	aminoglycoside antibiotics
Ab	Antibody
AChE	Acetylcholinesterase
AF	Aflatoxin
AMP	antimicrobial peptide
ATCL	Acetylthiocholine chloride
AuE	Gold electrode
B	Biochar
BC	Bacterial cellulose
BChE	Butyrylcholinesterase
BHL	(S)-N-butyryl-L-homoserine lactone
BIP	bacteria-imprinted polymers
BPNS	Black phosphorus nanosheets
BUHNPS	Bimetallic silver-gold sea urchin-like hollow nanoparticles
CA	Chronoamperometry
CB	Carbon black
CBZ	Carbendazim
CDs	Carbon dots
CFGO	Conjugated carboxyl graphene oxide
CFP	Chlorpyrifos
CFU	Colony-forming unit
CHI	Chitosan
CJ	*Campylobacter jejuni*
CNF	Carbon nanofiber
CNTs	Carbon nanotubes
COF	Covalent organic framework
CQDs	Carbon quantum dots
CRISPR	Clustered regularly interspaced short palindromic repeats
CS	Chondroitin sulfate
CV	coefficient of variation percentage
CV	Cyclic voltammetry
DA	Dopamine
DDVP	Dichlorvos
DFC	Diethofencarb
DMSO	Dimethyl sulfoxide
DON	Deoxynivalenol
DPV	Differential pulse voltammetry
dsDNA	Double-stranded DNA
E-AB	Methylene blue (MB)-modified aptamer
EDC	Entropy-driven amplification reaction
EFSA	European Food Safety Authority
EIS	Electrochemical impedance spectroscopy
ELISA	Enzyme-linked immunosorbent assay
ErGO	Electrochemically reduced graphene oxide
ERY	Erythromycin
Exo III	Exonuclease III
f-BN	functionalized boron nitride
FM	Fumonisin
g-C_3_N_4_	Graphitic carbon nitride
GC	Gas chromatography
GCE	Glassy carbon electrode
GLP	Glyphosate, N-(phosphonomethyl) glycine
GO	Graphene oxide
HCR	Hybrid chain reaction
hp DNA	Hairpin DNA
HPG	Highly porous gold
HPLC	High-pressure liquid chromatography
HS hpDNA	Hydrosulfuryl-modified hairpin DNA
IL	Ionic liquid
ITOE	Indium tin oxide electrode
IUPAC	International union of pure and applied chemistry
L	Leucine
L.M.	*Listeria monocytogenes*
LC–MS	Liquid chromatography- mass spectrometry
LOD	Limit of detection
LSV	Linear sweep voltammetry
mAb	Monoclonal antibody
MAL	Malathion
MB	Methylene blue
MC	Microcystin
Mfps	Marine mussel foot proteins
MIP	Molecularly imprinted polymer
MOF	Metal–organic framework
NEMA	Nicking enzyme-mediated
NPs	Nanopartcles
NRs	Nanorods
NW	Nanowire
OA	Okadaic acid
OPs	Organophosphate pesticides
OT	Ochratoxin
OTC	Oxytetracycline
PAs	Pyrrolizidine alkaloids
PAT	Patulin
PB	Prussian blue
PCR	Polymerase chain reaction
PDA	Polydopamine
PEF	Pefloxacin
PEI	Polyethyleneimine
PGA	Polyglutamic acid
PGE	Pencil graphite electrode
PLL	Poly(lysine)
PPY	Polypyrrole
PSA	*Pseudomonas aeruginosa*
PZDA	3,5-pyrazoledicarboxylic acid
QSS	Quorum-sensing system
R	Arginine
RBP	Receptor-binding phage
RBPP	Receptor-binding phage proteins
RCA	Rolling circle amplification
RPA	Recombinase polymerase amplification
RSD	Relative standard deviation
RT-qPCR	Quantitative reverse transcription polymerase chain reaction
SA	*Salmonella*
scMOF	Semiconductive metal–organic framework
SDZ	Sulfadiazine
SEN	Senecionine
SPAuE	Screen-printed gold electrode
SPCE	Screen-printed carbon electrode
SPE	Screen-printed electrode
SRCA	Saltatory rolling circle amplification
ssDNA	Single-stranded DNA
ST	*Staphylococcus aureus*
SWASV	Square-wave anodic stripping voltammetry
SWCNTs	Single-walled carbon nanotubes
SWV	Square-wave voltammetry
TA	Tannic acid
TBZ	Thiabendazole
TC	Tetracycline
TDN	tetrahedral scaffold
TICA	Trigging isothermal amplification
TM	Ti_3_C_2_ MXene
TMD	Transition metal dichalcogenide
TTX	Tetrodotoxin
UA	Uric acid
USFDA	United States Food and Drug Administration
VP	*Vibrio parahaemolyticus*
WHO	World Health Organization
β-CD	β-cyclodextrin
